# A Context-Assisted, Semi-Automated Activity Recall Interface Allowing Uncertainty

**DOI:** 10.1145/3770710

**Published:** 2025-12-02

**Authors:** HA LE, VERONIKA POTTER, AKSHAT CHOUBE, RITHIKA LAKSHMINARAYANAN, VARUN MISHRA, STEPHEN INTILLE

**Affiliations:** Khoury College of Computer Sciences, Northeastern University, USA; Khoury College of Computer Sciences, Northeastern University, USA; Khoury College of Computer Sciences, Northeastern University, USA; Khoury College of Computer Sciences and Bouvé College of Health Sciences, Northeastern University, USA; Khoury College of Computer Sciences and Bouvé College of Health Sciences, Northeastern University, USA; Khoury College of Computer Sciences and Bouvé College of Health Sciences, Northeastern University, USA

**Keywords:** Context-assisted Recall, Ecological Momentary Assessment, Wearable computing, Physical Activity Measurements

## Abstract

Measuring activities and postures is an important area of research in ubiquitous computing, human-computer interaction, and personal health informatics. One approach that researchers use to collect large amounts of labeled data to develop models for activity recognition and measurement is asking participants to self-report their daily activities. Although participants can typically recall their sequence of daily activities, remembering the precise start and end times of each activity is significantly more challenging. ACAI is a novel, context-assisted **AC**tivity **A**nnotation **I**nterface that enables participants to efficiently label their activities by accepting or adjusting system-generated activity suggestions while explicitly expressing uncertainty about temporal boundaries. We evaluated ACAI using two complementary studies: a usability study with 11 participants and a two-week, free-living study with 14 participants. We compared our activity annotation system with the current gold-standard methods for activity recall in health sciences research: 24PAR and its computerized version, ACT24. Our system reduced annotation time and perceived effort while significantly improving data validity and fidelity compared to both standard human-supervised and unsupervised activity recall approaches. We discuss the limitations of our design and implications for developing adaptive, human-in-the-loop activity recognition systems used to collect self-report data on activity.

## 1 INTRODUCTION

Accurate human activity detection could accelerate development of effective health interventions and interactive systems. Researchers in UbiComp, HCI, kinesiology, digital phenotyping, and behavioral science aim to leverage wearable sensing to build machine learning models for human activity recognition (HAR) [10, 17, 25, 56, 96]. Creating and validating such models, however, requires large sensor datasets with high-quality activity annotations. Often, researchers must collect datasets with people in the laboratory; these datasets may have limited label diversity and overly homogeneous signals, resulting in models that do not generalize to accurately recognize activity in varied and free-living contexts and across diverse individual lifestyles [21, 35].

The gold standard method for free-living data collection is to use an ego-centric camera [27, 33, 34, 48]. This approach presents several significant challenges. For researchers, video annotation is time-consuming, expensive, and susceptible to annotator bias. For study participants, on-body cameras create substantial privacy concerns, limiting recruitment and precluding deployment in extended studies [47].

Collecting participants’ self-reports is a more practical alternative for acquiring multi-day or multi-week annotation as people go about their normal lives. Two approaches to self-annotation are *momentary* and *retrospective* measurement. A popular momentary approach is ecological momentary assessment (EMA) [95], where participants respond to surveys prompted on mobile or wearable devices [4, 23]. EMA, however, induces interruption burden, creating contextual response biases (i.e., participants’ receptivity to the prompt heavily depends on their contexts) that generate data missingness and label imbalance, ultimately compromising the quality of the dataset [29, 38, 50, 68, 73, 81]. Modifications to EMA, such as “microinteraction” EMA (*μ*EMA), where single-question prompts are delivered via accessible wearables can collect high-density activity data at 5–15 minute intervals [41, 60], may reduce burden under some data collection conditions.

*Retrospective* methods address these limitations by allowing participants to report activities by recalling them after they are completed, typically at the end of the day [10]. This approach reduces interruption burden and data missingness while enabling more natural annotation behaviors. Retrospective recall can be conducted with or without human supervision [14, 115]. The gold standard for 24-hour activity recall is the 24PAR (24-hour Physical Activity Recall) method, a validated, structured, human-administered physical activity assessment instrument that collects details about study participants’ past-day activities [72]. In a 24PAR interview, the interviewer probes in real-time, helping participants to remember short-burst activities and filling in gaps in a daily activity timeline. This method has a low cognitive burden on participants and has been used with all age groups [115]. Researchers, however, face significant scalability challenges in administering the 24PAR because it must be administered daily by trained staff, thus limiting the number of participants that can be enrolled simultaneously and increasing administration cost. Furthermore, the 24PAR is time sensitive and requires that interviews be scheduled within a narrow window every day, making coordination with participants challenging and often leading to missed appointments and delays.

Digital recall methods—administered through a website or a mobile app—offer a more scalable and efficient alternative to 24PAR. These tools allow participants to self-report at their convenience, reducing the burden on researchers to schedule and conduct interviews [14]. Digital recall methods also enable real-time data collection, automatic data synchronization, and standardized prompts, which help improve data consistency and reduce interviewer bias. Despite these advantages, digital recall methods still induce recall bias, especially regarding activity start and stop times. Participants who incorrectly recall the start or end time of one activity may create systematic errors, distorting the timing of all subsequent activities and potentially skewing the entire timeline [95]. Automatic context-assisted recall can mitigate recall bias by providing participants with contextual cues that can help them better remember their activities. These cues can include physiological data (e.g., heart rate), location data, or environmental triggers (e.g., weather or sounds) that may be linked to the activities participants perform. By providing participants with such context, they may be able to more accurately pinpoint the timing of their prior activities [76, 86].

Even using automatic context-assisted recall, precise identification of activity transition times, i.e., the moment when one activity ends and another begins, can be difficult because activity boundaries are often not clear-cut. For example, the transition from “walking” to “lying down” may not occur at a single moment; it may involve a gradual change in behavior and posture. In addition, interleaved activities, where one activity overlaps with another, further complicate the task of indicating precisely when activities begin and end. Misremembering or misreporting these transitions can create errors that propagate through the entire recall, distorting the timeline. When using labels for training and validating HAR algorithms, researchers have shown that even a 5% misalignment in activity boundaries can lead to a significant drop in model performance [57]. Nevertheless, despite the difficulty and inherent uncertainty of the labeling task, most context-assisted recall methods and interfaces do not allow participants to express this *temporal uncertainty*.

Although unsupervised digital recall methods offer efficiency by allowing participants to generate their own activity annotations, they often lack the nuanced probing that human supervision provides. In a 24PAR interview, interviewers can ask follow-up questions, clarify vague responses, and prompt participants to recall additional details about their activities, leading to more consistent and accurate data. In contrast, unsupervised methods rely on the participant’s ability to recall and annotate their activities independently, which can result in significant variability in the quality and completeness of annotations across participants and also within participants over time [103]. Some participants may provide highly detailed and accurate annotations, while others may produce sparse or inconsistent labels, leading to inconsistencies across the dataset. A semi-supervised method that generates label suggestions based on passively sensed data, allowing participants to “fix” or “confirm” these suggestions instead of creating new annotations from scratch, may combine the scalability and convenience of digital recall with the human oversight necessary to ensure consistent and high-quality annotations.

To gather ground-truth activity labels from people in free-living environments, researchers must develop ecologically valid data collection systems that allow individual participants to accurately self-label their activities, ideally expressing temporal uncertainty. To sustain such labeling for a week or more, such systems must maintain participant engagement with a manageable cognitive workload. To assist people with labeling, such systems may need to combine momentary labeling and automatically acquired contextual cues.

We present ACAI, a novel semi-automated annotation system that allows participants to self-annotate their activities up to a five-minute granularity. In ACAI, a user interface presents people annotating their own activities with system-generated activity blocks pre-filled with activity predictions; study participants can “fix” or “confirm” these predictions rather than creating annotations from scratch. Additionally, the ACAI interface provides participants the option to label time intervals during which they are not entirely certain about their activities as *“uncertain intervals.”* We hypothesize that the design of ACAI will reduce participant burden while producing higher-validity annotations obtainable during week-long or multi-week field studies.

We address the following research questions:

- **RQ1:** What are the effects of uncertainty annotation and system suggestions on system usability, annotation time, and participants’ perceived workload?
- **RQ2:** What is the criterion validity of the annotations generated by users using our annotation system?
- **RQ3:** What is the feasibility of using our proposed annotation interface in a week-long free-living study?

## 2 BACKGROUND

Human activity recognition (HAR) refers to the problem of identifying specific activities or postures using wearable sensors. Researchers can use activity labels collected in free-living settings to train activity recognition models, or simply to understand a person’s daily habits and lifestyle. Large data sets with accurate activity labels that are synchronized with sensing data lead to better model training and a more informative evaluation process. However, training data sets for human activity recognition are often collected in heavily controlled [5, 19] or semi-controlled [21] laboratory settings. This leads to limited sets of labels and behaviors that may not reflect the complexity of the same behaviors in free-living environments. Models trained on such datasets often fail to generalize, performing poorly in less-controlled real-world scenarios [85] where people have a wider range of activities or experience drastic changes in mobility patterns or lifestyles that can impact the distribution of the recorded signals.

There have been attempts to capture information on a person’s free-living physical activity (PA) labels in the wild to support better training of HAR models and more realistic evaluation. There are two major label collection methods: *momentary* vs. *retrospective*.

### 2.1 Measuring activities in-the-moment

One method of acquiring *momentary* activity labels is direct observation, where participants wear egocentric cameras to capture time-lapse images or videos that are later manually annotated by trained coders [33, 48]. Privacy concerns, however, limit the use of this approach in multi-day studies, and annotation is labor-intensive. Annotation quality also depends heavily on the annotators’ ability to infer activity from first-person views without participant input.

An alternative is self-report. Ecological momentary assessment (EMA), or experience sampling, involves prompting participants via mobile or wearable devices to report activities in real time [92, 95]. While widely used in research studies, EMA can be intrusive, making frequent prompts burdensome and reducing compliance. As a result, researchers must balance frequency and burden to maintain response rates, limiting temporal resolution. *μ*EMA [41] addresses this by using 2–3 second smartwatch prompts, enabling high-frequency labeling (e.g., four times per hour) while maintaining compliance [80, 81]. This method is constrained by screen size, cognitive simplicity (e.g., less than four response options), and requires two-hand interaction. Audio-*μ*EMA supports speech input for open-ended, hands-free labeling and enables broader behavioral coverage while sustaining high response rates [60]. All EMA methods may still suffer from contextual non-response bias [61, 81], affecting the diversity of data captured.

Because activity labels require accurate start and stop times—which EMA prompts do not easily provide [52]—designs like START/STOP allow users to manually mark activity boundaries [18, 106], similar to systems used by Fitbit [31] and Strava [97]. This method, however, can feel unnatural and burdensome, requiring users to pull out their phone or watch and remember to stop the recording [104]. Weppner et al. introduced a smart glasses interface where head or finger gestures marked activity boundaries [109]. The extraSensory app prompted users to indicate both the start time and expected duration of an activity [103], but interruptions or changes in behavior can still result in inaccurate labeling. Although the main purpose of their design was to enable accurate segmentation of activity boundaries, this approach can fail if participants forget to click the STOP button when they finish, or overestimate the expected duration of the activity.

### 2.2 Recalling activities retrospectively

Another common method to collect activity labels in free-living settings is to use a recall interface, where participants input their activities at the end of a day, after the activities have concluded. Retrospective recall eliminates interruption burden and activity interruption. Schroeder et al. proposed an interface that allowed participants to annotate using a web application by choosing the assigned tags of the activity and its start/stop time [88]. Khan and Maes designed an interface that used a clock’s circular shape to allow participants to indicate the start and end times of a label [49]. Mairittha et al. presented a conversational mobile interface that allowed participants to generate labels using speech [71]. This approach is scalable to earables or smartwatches to mitigate the need for participants to pull out the phone, thus creating a hand-free interaction that is less disruptive to participants.

One downside of using *retrospective* recall interfaces is recall bias, where events happening before or after an event affect the recall of the event. Fast-changing, overlapping sequences of activities [8] can lead to mistakes in labeling activity boundaries, which can significantly reduce model performance [57]. *Context-assisted* recall presents participants with additional information/context that may jog their memories. Dunton et al. presented a mobile application that allowed participants to annotate their physical activity with the help of accelerometer sensing data [28]. Rabbi et al. allowed participants to annotate their physical activity along with their food and take pictures of their food to assist with recall [87]. Hoelzemann and Laerhoven compared the effectiveness of models trained on datasets collected using in-situ measurements and a desktop retrospective recall interface with visualization of accelerometer data (time-series recall) and found that models performed better on the time-series recall dataset [39]. Korpela et al. presented a desktop interface that showed both the accelerometer signals and the output of a depth camera on the screen to facilitate better segmentation of labels [55].

### 2.3 Human-AI collaborative annotation system

Human-computer collaboration has been a long-standing topic in HCI research. Achieving effective human-AI complementarity, however, is not guaranteed and requires careful design. Recent studies show that human-AI teams can sometimes perform worse than either humans or AI alone across various tasks [102]. A key barrier to successful collaboration is improper calibration of trust—specifically, under-reliance (ignoring helpful suggestions) and over-reliance (accepting incorrect suggestions) on AI systems [9, 13, 45, 58]. These issues highlight the need for systems that support better coordination between human judgment and AI suggestions.

Humans and AI often have distinct and complementary strengths. When users are trained to understand these differences, they can make better decisions about when to rely on the AI and when to take control themselves [69, 70, 78]. This complementary approach is especially valuable in annotation tasks, where human contextual knowledge can refine or correct AI-generated predictions.

There has been growing interest in human-AI collaborative annotation, spanning domains such as document [30], video [26], image [93], audio [7], and biomedical [11] data. More recently, AI-generated labels have been integrated into annotation systems for passive sensing data. For example, Stojchevska et al. allowed participants to review and edit predicted activity labels at the end of the day [99], and extraSensory enabled users to refine model-generated labels [103]. Similarly, Neupane et al. showed how reviewing context and stress predictions can improve labeling accuracy [76]. These studies have demonstrated that incorporating AI suggestions can reduce cognitive burden and accelerate the annotation process [26].

To address the limitations of current retrospective annotation systems—particularly challenges related to recall bias and temporal uncertainty—we introduce ACAI, a context-assisted annotation interface that supports multi-label activity annotation at five-minute granularity. ACAI leverages passive sensing data to generate activity suggestions that participants can either “confirm” or “correct.” ACAI also allows participants to mark *uncertain* intervals, enabling the expression of human uncertainty in the annotation process.

## 3 ACAI: SYSTEM DESIGN AND RATIONALE

We present the components of ACAI and the design rationale behind each component. In Section 3.2, we describe the hypotheses that drove our interaction design based on prior research, and we describe the implementation of three different versions of the interface that were used in the user study.

### 3.1 Visualization of contextual cues to assist recall on mobile interface

**Fig. 1.**
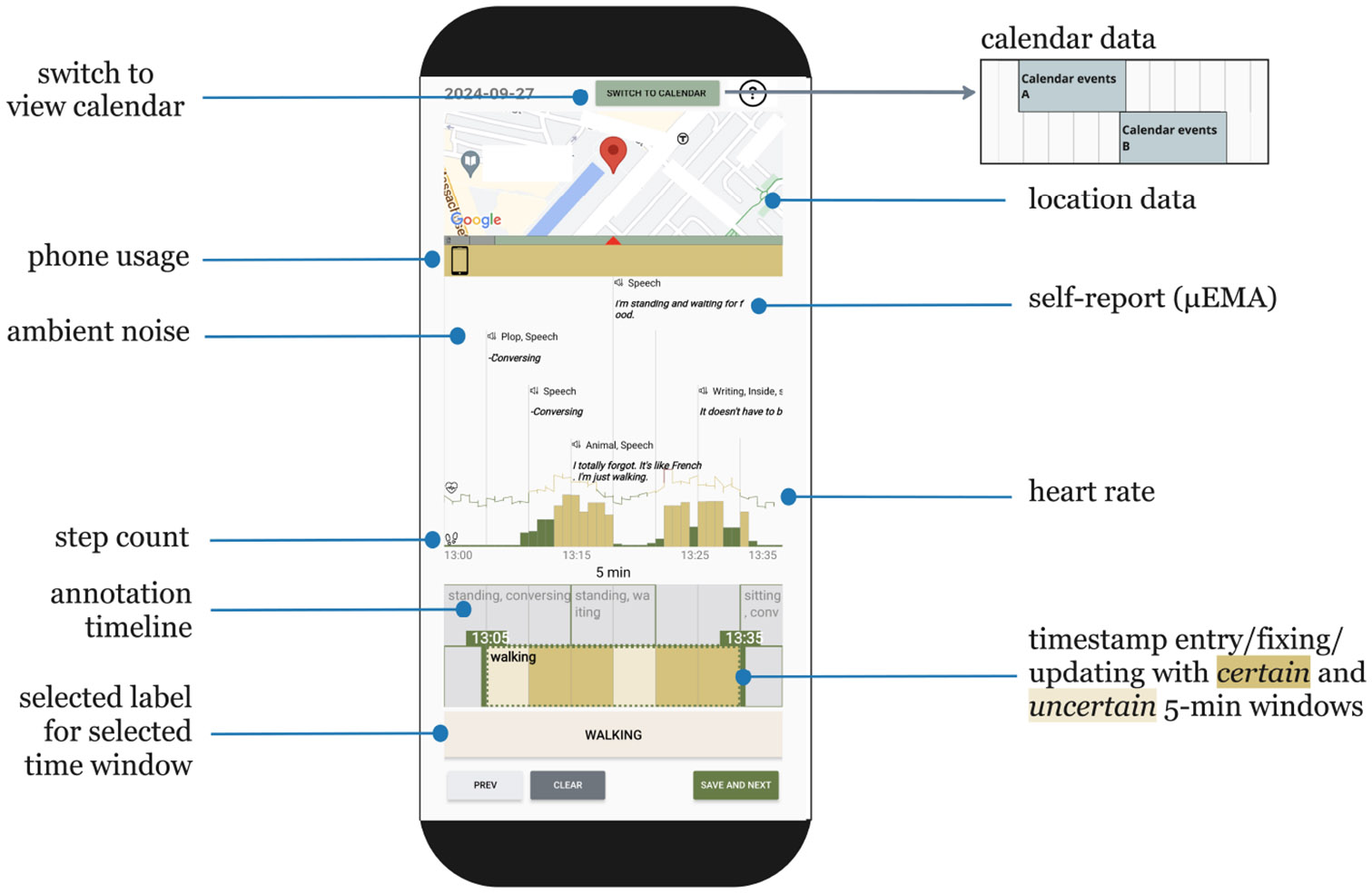
Mobile interface of ACAI. The top two-thirds of our annotation interface presented participants with their self-reports and passively collected contextual cues to aid with the recall process. Users could switch between a location view and calendar view. The bottom third displayed the annotation timeline and labeling controls.

The top two-thirds of the ACAI display showed six different passively collected contextual cues, along with *μ*EMA responses: location, ambient noise, calendar events, heart rate, phone usage, and step counts. All contextual cues were displayed within the screen of ACAI so that users did not need to scroll between different panels. A screenshot of the visualization panel in Figure 1 shows:

- *Location data/Calendar events:* Location data (GPS) were collected once every minute from the participant’s phone. When LOCATION was selected, the location for the current time on the activity timeline was displayed at the top of the screen on a Google map. As the activity timeline moved forward or backwards in time, the location changed. When CALENDAR was selected, calendar events retrieved from the participant’s Google or Outlook calendar were shown. Participants could click on the “SWITCH TO CALENDAR” or the “SWITCH TO LOCATION” button at the top of the screen to toggle between these views.
- *Phone use:* The mobile application logged the phone status (i.e., whether the phone screen was on and/or unlocked) once each minute, and the phone use status was displayed as a heat map below the location/calendar view. A yellow interval indicated that the phone was being used, and a white interval indicated that the phone screen was off.
- *Step count:* Step count was measured by the smartwatch once every minute, and the data were displayed below the phone use view. Because a vertical axis is space-consuming horizontally, and displaying numbers on top of the bars can lead to information overload and confuse users, we color coded the value of the step count. Green bars indicate small movement or sedentary (<50 steps per minute), yellow bars indicate slow walking to normal walking (50-150 steps per minute), and red bars indicate brisk walking to running (>150 steps per minute). The threshold was chosen based on prior work on the young adult population [101], and we adapted the threshold mentioned in the paper based on our internal testing of the Pixel Watch.
- *Heart rate:* Average heart rate data from the watch were displayed as a line graph with the step count data. Although heart rate varies with age, physical activity level, and lifestyle, given the homogeneous population in our user study (young and emerging adults), we categorized heart rate as resting (color-coded green), moderate (color-coded yellow) and vigorous (color-coded red). The threshold was chosen based on prior work (formula based on age) [100], and we adapted the threshold mentioned in the paper based on our internal testing of the Pixel Watch.
- *Ambient noises:* Every five minutes, the application ran Google’s YAMNet noise classification [32] on 10-second audio clips. No raw audio clips from this process were stored on the device but the classification ouptut was transferred to the phone and displayed above the heart rate and step count.
- *μEMA responses:* Once every 10 minutes, participants were prompted on the watch using *μ*EMA. The audio clips collected from speech-based *μ*EMA were analyzed using a fine-tuned Google Cloud speech-to-text v1^1^ model running on the watch; the results of the speech recognition were transmitted to the phone once every hour. The self-report activity labels were displayed as bolded text in the middle section of the screen, along with the ambient noise classification results.

### 3.2 Interaction design

In this section, we describe the interaction design behind our system. Our approach was guided by two main hypotheses:

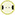
 H1: An appropriate interaction design that allows participants to express uncertainty will not increase their perceived burden or the time they believe annotation takes.

Although recalling an activity might be relatively easy, recalling the exact start and end times of an activity might be considered a more challenging task [74]. This recall challenge makes annotating activity transitions unreliable and can cause systematic errors if the recalled duration or activity timestamps differ significantly from the actual activities [57]. We hypothesize that a well thought-out interaction design that can allow participants to express their temporal uncertainty about the start and end time of an activity will not increase annotation time, perceived effort, or adversely affect system usability.

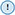
 H2: Participants will find it easier to modify system-suggested annotations than to add new annotations manually.

Recently, multiple research teams have investigated human-AI collaboration systems, specifically systems where the users review the AI-generated suggestions and make changes [107]. These studies show that providing users with AI predictions to review reduced the time and effort for users in multiple tasks (e.g., writing, programming, creativity, planning) [43, 62, 63, 113]. In this study, we hypothesized that a well-designed interface that allows participants to adjust/fix the suggested annotation would increase the usability of the system and the speed of annotation.

Given our iterative design process, we decided to test our hypotheses on the utility of the two major components as we developed ACAI. Thus, we implemented three different versions of the annotation interface, with three different combinations of functionalities presented by ***H1*** and ***H2***. We conducted a study where we asked 11 participants to collect two days of data in the free-living settings followed by an in-lab usability session where participants interacted with the three prototypes of annotation interface using their own data. This helped us collect participants’ preferences and evaluate our hypotheses. To keep the time required for the in-lab session manageable and to avoid participants annotating the same data multiple times, potentially causing data skewness, we decided to limit the in-lab evaluations to the three versions of the interfaces that allow us to test our hypotheses, and not all combinations of all features. We also wanted to ensure that participants took roughly the same amount of time to annotate on each of the three versions. For each version, there are three main supported interactions: *adding, editing*, and *deleting* annotations.

**Fig. 2.**
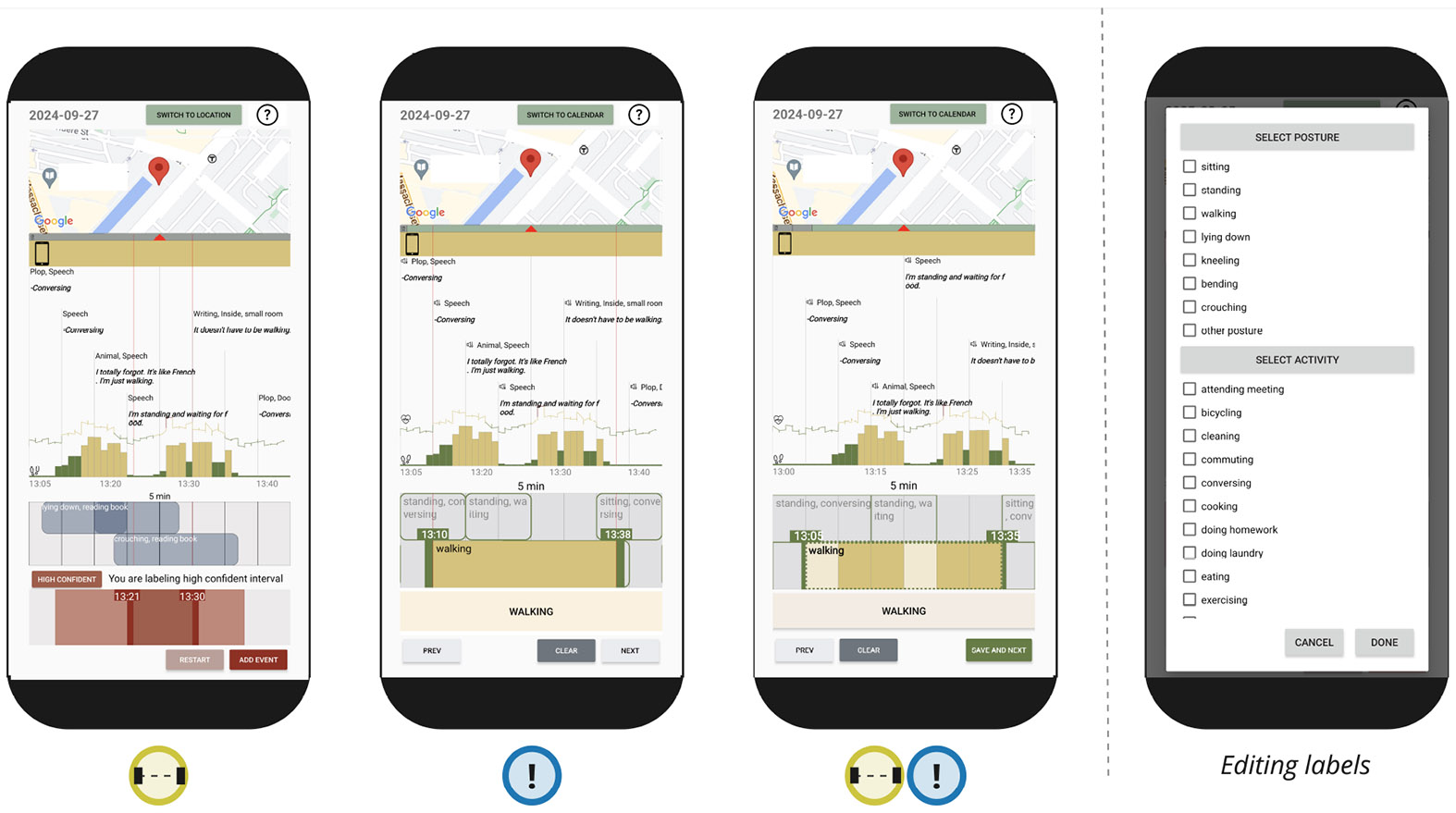
Three different versions of interactions in ACAI were evaluated. Version 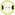 allowed uncertainty labeling but did not make automatic annotation suggestions. Version 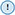 made automatic annotation suggestions but did not allow uncertainty labeling. Version 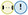 had automatic annotation suggestions and allowed users to indicate labeling uncertainty. Labels were selected from a list of postures and activities.

In version 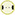, participants could add the timestamps of the certain and uncertain intervals by sliding the anchors representing the start and end of each activity interval. In version 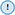, there were no uncertain intervals. Participants fixed the timestamps of the system suggestions by sliding the anchors representing the start and end times. In version 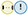, participants could tap-and-slide a finger through the timeline to flip the status of each time block from “no label” → “uncertain” → “certain.” In all three versions, participants could add/fix the posture/activity of the label by tapping on a drop-down menu (see Fig. 2). To edit an annotation, in all three versions, participants could long-press on the targeted annotation and adjust the timestamp and the label in the same way as when *adding* a label. To delete an annotation, participants could long press on the targeted annotation then and click on the DELETE button (or CLEAR in version 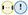).

We also supported additional interactions to navigate the timeline. These included *scrolling, zooming in* and *zooming out*. Participants could scroll forward or backward on the timeline by sliding a finger left/right on the screen. Participants could adjust zoom levels by double tapping or pinching two fingers on the screen. In version 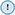 and version 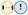, the timeline auto-scrolled and zoomed when participants moved to a new suggestion (clicking NEXT/PREV), centering the current activity block on the screen. We expected this auto-scroll and zoom would reduce participant’s manual navigation time.

Comparing version 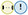 with version 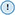 enabled us to study the effects of “uncertain” labels (Hypothesis 1); and comparing version 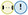 and version 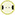 enabled studying the efficacy of system-suggestions on participants’ burden and annotation behaviors (Hypothesis 2). Additional details about the interaction design of ACAI are available in Appendix B.

#### 3.2.1 μEMA interactions.

We used the same multimodal-*μ*EMA system proposed by Le et al. [61], adjusting the prompting interval to once every 10 min. This *μ*EMA system was shown to be usable and have high compliance when deployed in a week-long field study. Participants were prompted to report their in-the-moment posture and/or activity, and they could respond in one of three ways: 1) by tapping on activity options displayed on the watch screen, 2) by writing letters on the watch screen to narrow down activity options that were displayed, or 3) by speaking the current label to the watch.

### 3.3 Implementation

**Fig. 3.**
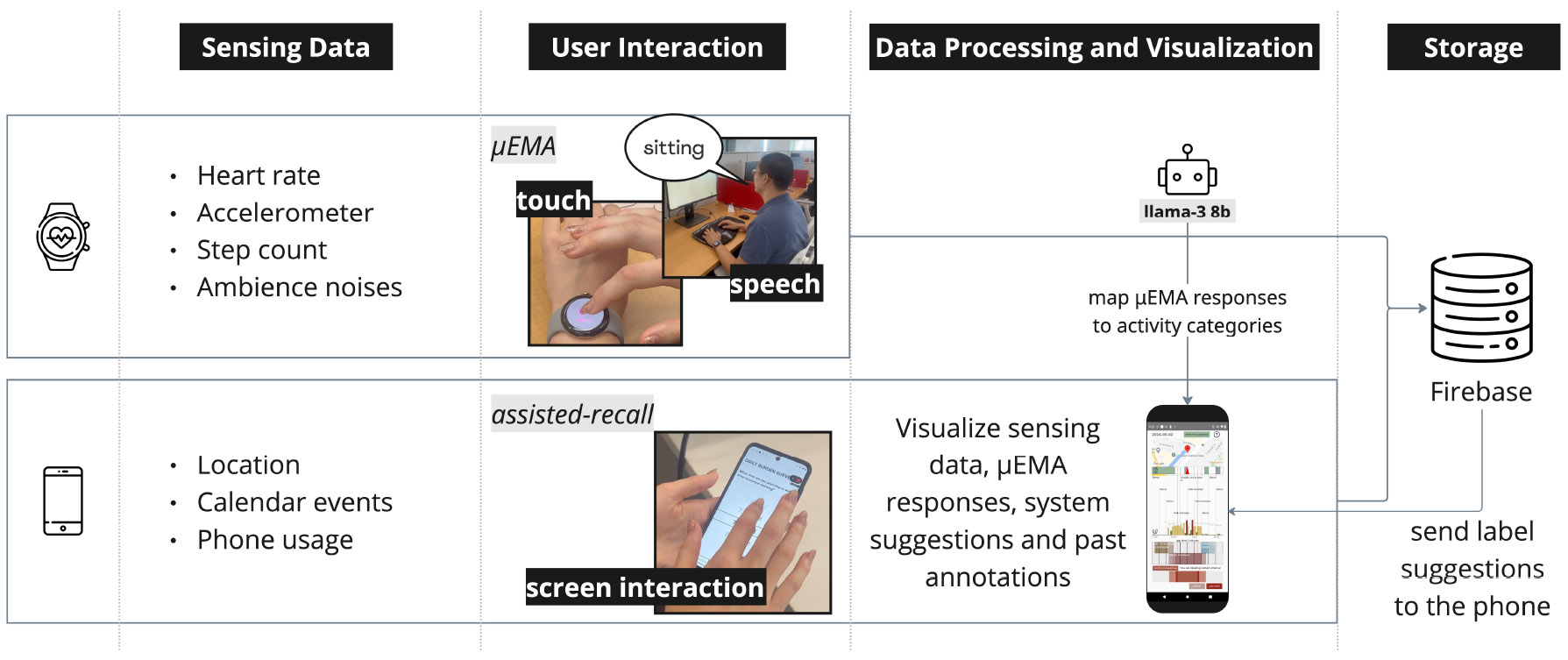
Overview of our smartphone-smartwatch system.

We implemented and deployed our system on the Android operating system. Our mobile application runs on all Android phones running Android 9 or later, and the WearOS application runs on the Pixel Watch 2. We installed the app on participants’ personal phones. We show an overview of our system in Figure 3. The phone and the smartwatch continuously collected passive sensing data in the background and transferred the data to our Firebase server every hour throughout the study period. We stored and processed the passive sensing data to generate system suggestions of posture and activity. Approximately one hour after context-cue data were collected by the watch and phone, suggested activity labels appeared in the phone ACAI interface. We present the details about how the algorithm generated system suggestions in Appendix E.

We chose to implement ACAI on a mobile platform rather than a web interface for two main reasons. First, mobile apps allow participants to conveniently annotate their activities throughout the day, which can improve compliance and recall accuracy by reducing the delay between the activity and annotation. Although web interfaces on laptops may offer more fine-grained interactions, they are less practical than mobile devices for participants to carry and use on the go. Second, a mobile app reduces data latency. A web-based interface would require transferring data from the watch and phone to a remote server via the internet, which may not always be available. In contrast, a mobile app can receive data directly from the watch over Bluetooth, enabling lower-latency and more reliable real-time annotations.

## 4 STUDY DESIGNS

We conducted two studies with a total of 24 participants. The first study with 11 participants was a usability evaluation in which we wanted to understand the effects of annotating uncertainty and auto-suggestions on system usability, annotation time, and effort (Hypothesis 1 and 2). We conducted the second study with 13 participants over two weeks. The goal was to evaluate two aspects of the system: in the first phase (week 1) the aim was to assess the criterion validity of our annotation interface, and in the second phase (week 2) the aim was to evaluate the longitudinal usability and sustainability of the interface in free-living settings.

### 4.1 Recruitment and participant compensation

Our study protocol was approved by the institutional review board at Northeastern University. Participants were recruited via emails to relevant mailing lists, social media posts, and posters placed around the university campus. We invited interested participants for an initial Zoom call to determine eligibility. Eligibility criteria included participants 1) aged 18 or above, 2) who had no cognitive, visual, or hearing impairments, 3) were willing to wear a smartwatch for up to 14 days, 4) were willing to wear a motion sensor taped to their thigh for up to seven days, and 5) had an Android phone and were willing to download an Android application on their phone. During the initial Zoom call, a research assistant went through the consent form with the participants and asked them to download an Android application through the Google Play Store to check their device’s eligibility (memory, storage, and sensor availability). We compensated participants $50 for Study 1 and $110 for Study 2 via Amazon gift cards.

Both studies followed a within-subject design with three conditions. We calculated the required sample size for the study using a one-way repeated-measures ANOVA test with *α* = 0.05, power = 0.8, number of conditions = 3, *ρ* = 0.5, *ϵ* = 1.0, and *η*^2^ = 0.14 (large effect size), resulting in a sample size of *n* = 11 participants. We recruited 11 participants for the first study, and 14 participants for the second study. One participant participated in both studies. We list participant demographics in Table 1; all participants in our studies were students at Northeastern University.

### 4.2 Introductory session

We invited eligible participants to an in-person introductory session. A research assistant obtained signed consent from the participants and guided the participants through the setup of the application on their phones. The research assistant paired a Pixel Watch 2 to the participants’ phones and downloaded the WearOS application on the watch. If a participant frequently used Outlook calendar to log events or meetings, the research assistant asked for permission to sync the participant’s Outlook calendar with the Calendar application on their phone. This allowed our system to access the participant’s calendar events in real time.

In both studies, we asked participants to complete a survey about demographic information, physical activity level, and familiarity with self-tracking tools (e.g., fitness trackers, smartwatches). The research assistant gave an introduction to the functionalities of the mobile annotation interface and showed an example of how the data would be represented on the interface. We trained participants to answer *μ*EMA questions on the watch using a ten-minute tutorial video (see Supplementary Materials). In the second study, we also instructed each participant on how to attach a research-grade Actigraph GT9X Link sensor (Actigraph, LLC) to the top of the middle of the thigh directly on the skin with medical adhesive tape; the sensor application was waterproof and the sensor could be worn continuously for a week. Participants could choose to use the left or right thigh.

### 4.3 Study protocols

The first study was a usability study in which participants collected data in their natural environments using a provided smartwatch and their personal phone for two days; they also attended a one-hour in-lab session. The second study was a two-phase evaluation, where each phase lasted for one week, during which participants collected data and used the annotation interface in free-living settings.

#### 4.3.1 Study 1: Iterative design and comparison.

In Study 1, we aimed to answer **RQ1 – What are the effects of uncertainty annotation and system suggestions on system usability, annotation time and participants’ perceived workload?** We conducted a usability study to compare three different versions of ACAI. We instructed participants to wear watches, carry their personal phones, and answer *μ*EMA prompts for two days. Participants did not interact with the phone app or the annotation interfaces during the free-living portion of Study 1.

At the end of the two-day free-living portion of the study, we invited participants to the final usability session and exit interview. We introduced participants to three versions of the annotation interface (Figure 2). For each version of the interface, participants completed a tutorial built into the application and learned how to annotate their posture and activity. Each tutorial ended with a series of tasks to test participants’ understanding of the system. Participants could ask the research assistant in the room questions during the tutorial as needed. After the tutorial, participants used the interface to annotate one day of posture and activity (of the two days in the free-living portion of the study) in a think-out-loud session. The same day was used for all the interfaces version, and the order of the interfaces was randomized for each participant to minimize carryover effect. Participants then completed a usability survey assessing each interface version—the survey consisted of an adapted version of the system usability scale (SUS) [12] and the NASA-TXL scale [15] (see questions in Table 5, Appendix A). We asked participants open-ended questions at the end of the session about which features they preferred, which version of the interface was most intuitive to them, and their overall experience collecting data in the field.

#### 4.3.2 Study 2.1: Within-subject validity study.

The first phase of Study 2 aimed to answer **RQ2 – What is the criterion validity of the annotations generated by users using our annotation system (compared to current practice)?** To evaluate this question, we compared the criterion validity of the annotations produced by ACAI against two standard recall methods: a *standard 24-hour physical activity recall* (24PAR), and a *computerize 24-hour activity recall* (ACT24). Participants used each method for two days (in total six days for three conditions), and we counterbalanced the order of the conditions to account for potential order effects and minimize bias across participants.

The 24 Physical Activity Recall (24PAR) is a telephone-and-interviewer administered self-report instrument designed to collect information about activity duration, energy expenditure, and the context of an individual’s physical activity and sedentary behaviors throughout the day [53]. During the 24PAR, the interviewer asks participants to report on their activities in the past 24 hours in bouts of 5 minutes. The interviewer can ask follow-up questions during the 24PAR to extract detailed annotations from the participant. The reported activities are matched to the corresponding MET score from the Compendium of Physical Activity (CPA) [36]. Prior research has shown the robustness of 24PAR against objective measurements (such as the SenseWear Armbands [53, 98] or a hip-worn sensor [46]). Administering the 24PAR, however, is time-consuming for both participants and the research team, making it difficult to scale for larger studies. In the 24PAR condition, a member of the research team set up an online call with the participant each morning to complete a 24PAR recall interview. The call lasted about 30 minutes. We asked participants to recall their activities and postures from the day before.

Due to the difficulty scaling 24PAR, researchers have developed Activities Completed over Time in 24 Hours (ACT24), a digital web-based version of the 24PAR [37]. We used the third version of the software for our study (ACT24 v3.0), which is compatible with multiple devices including tablets, smartphones, desktops [46]. Multiple studies have confirmed the validity of ACT24 against objective measurements, including activPAL and SenseWear Armbands [46, 98]. In the ACT24 condition, we sent a message to each participant’s phone number and email address each morning with a link to their ACT24 accounts. Participants recalled and annotated their activities and postures of the day before using the link.

In the ACAI condition of Study 2.1, we instructed participants to wear the watch, carry their personal phone, and answer *μ*EMA prompts throughout the day. Each participant received two prompts on their phone every day—one at noon and one at 8 pm—to use the app to annotate their activities.

Additionally, we asked participants to wear the ActiGraph GT9X Link sensor on their thigh for six days, even during sleep. We used the 80 Hz (range ±8 g) tri-axial accelerometer data from the thigh sensor as the objective passive sensing measure to assess the validity of the three annotation mechanisms [46, 52, 98].

#### 4.3.3 Study 2.2: Feasibility study.

Phase 2 of Study 2 aimed to answer **RQ3 – What is the feasibility of using our proposed annotation interface for longer study duration (seven days)?** Immediately after the six days in Study 2.1, we asked participants to continue using our proposed annotation interface for another seven days. We measured whether participants’ annotation behaviors changed over time and evaluated the ability of ACAI to collect diverse and naturalistic activity patterns and habits. In this phase, participants did not need to wear the ActiGraph thigh sensor. Although the GT9X Link sensor can be worn comfortably on the thigh for about a week, after a week the medical tape can begin irritating the skin and become uncomfortable.

After the end of the 13-day period, we invited participants to an in-person exit interview. During the interview, participants attended a semi-structured interview with a member of the research team. Participants shared their overall impression and experience with the system and provided suggestions on improving the implementation the annotation interface. Each session lasted approximately 60 min, and we recorded the audio of the session after obtaining consent from the participants.

## 5 FINDINGS FROM THE USER STUDIES

In this section, we present our findings from the two user studies, following the respective research questions.

### 5.1 Impacts of uncertainty and auto-suggestions on system usability, annotation time, and perceived workload (RQ1)

We present the results from Study 1 with 11 participants, based on the hypotheses that drove our design decisions.

**Table:** 

H1: An appropriate interaction design that allows participants to express uncertainty will not increase their perceived burden or the time they believe annotation takes.
We found no statistical differences in annotation duration, system usability, and perceived efforts between version 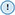 and 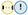 (*p* > .05).

**Fig. 4.**
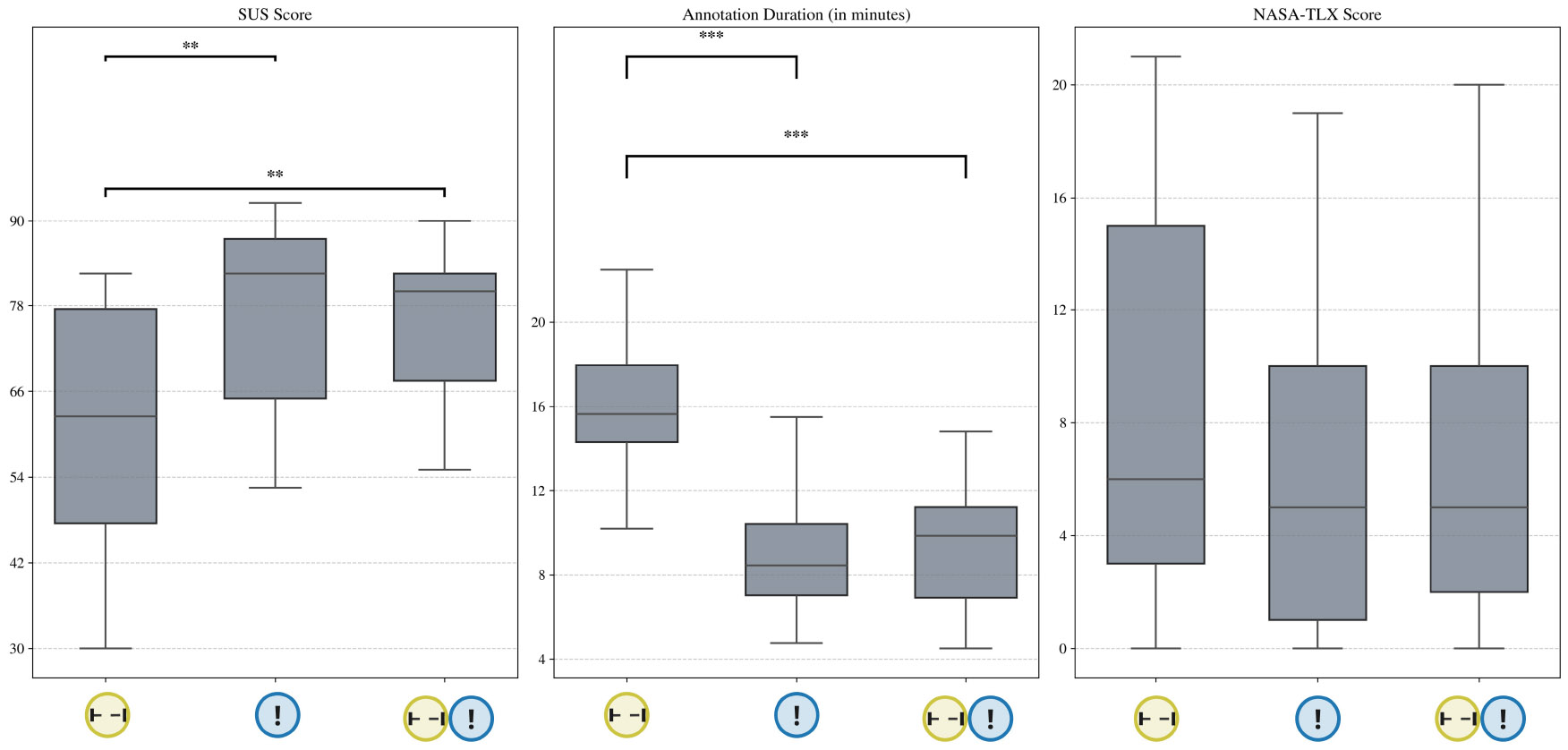
System usability score, annotation time, and NASA-TLX score across three conditions in Study 1. *** p-value < .001, ** p-value < .05.

Overall, we found no differences in usability, annotation time, and perceived effort between version 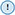 and version 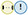. We show the distribution in system usability score, annotation time, and perceived effort (NASA-TLX score) across the three conditions in Figure 4. Asking participants to annotate certain and uncertain labels did not decrease quantitatively assessed system usability or increase annotation time. The semi-structured interviews, however, uncovered some differences in participant perceptions. Of the 11 participants in the study, five expressed that having uncertainty intervals was helpful for annotation: *“ I think the uncertainty is helpful because […] I could not recall everything that I did. So it’s better to have that than [not]” [U3]*. Five other participants, however, commented that adding the uncertainty intervals increased their recall efforts. They attributed the burden to 1) the additional physical effort (i.e., time) to add an additional interval with uncertainty, and 2) the additional mental effort required to assess their confidence in the annotations.

Referring to the time taken to annotate uncertainty in the interval, one participant said, *“It complicates things too much. I think having two different certainty uncertainty takes too much time and I don’t know how this will be used” [U2]*. Three participants expressed that the physical effort might be reduced by a redesign of the interaction, one saying *“Doing the uncertainty part is annoying, especially in the first [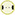] option. But the way we could do it in the second [*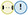 *option] was much better just by tapping where you could. It’s very fast, if you had to put uncertainty, that was the way I would rather do it and [another way when I have to drag the intervals twice] it’s very difficult” [U11]*.

Participants also mentioned the mental effort associated with adding uncertain intervals: *“I feel like switching between uncertain and certain was a little mentally demanding itself” [U6]*. This mental effort can arise from the participants’ interpretation of uncertainty: *“Because I feel like there is no way for me to accurately remember what I did every minute. The voice input (μEMA) was very helpful, but still, I can’t remember which minute I was doing something. So even if I can input like, I was certain for this period, I was doing this [activity]. I was not 100% certain anyways” [U6]*. Two participants also said that adding an uncertain interval forced them to think more carefully about the timestamp: *“[To annotate] separately the uncertain part and the certain part, I have to recall more carefully” [U11]*.

**Table:** 

H2: Participants will find it easier to modify system-suggested annotations than to add new annotations manually.
Pairwise comparison with Tukey adjustment shows that the system usability score of version 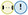 is significantly higher than version 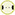 (*t* = 4.96, *p* < .001). Pairwise comparison with Tukey adjustment shows that the annotation time for version 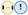 is significantly lower than version 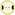 (*t* = −2.73, *p* < .001). We found no difference in perceived effort between version 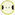 and 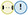 .

All eleven participants commented that the system-suggested labels were helpful and improved annotation speed. Participants preferred the automatic scroll and zoom function between segments: *“I like the next and the previous (auto-scrolling) functions. So the moment you are done […], it just automatically went to the next. That was helpful” [U1]*. We show the interaction counts for each ACAI version in Table 2; participants engaged in significantly more manual scrolling in version 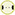 than the other two versions.

**Fig. 5.**
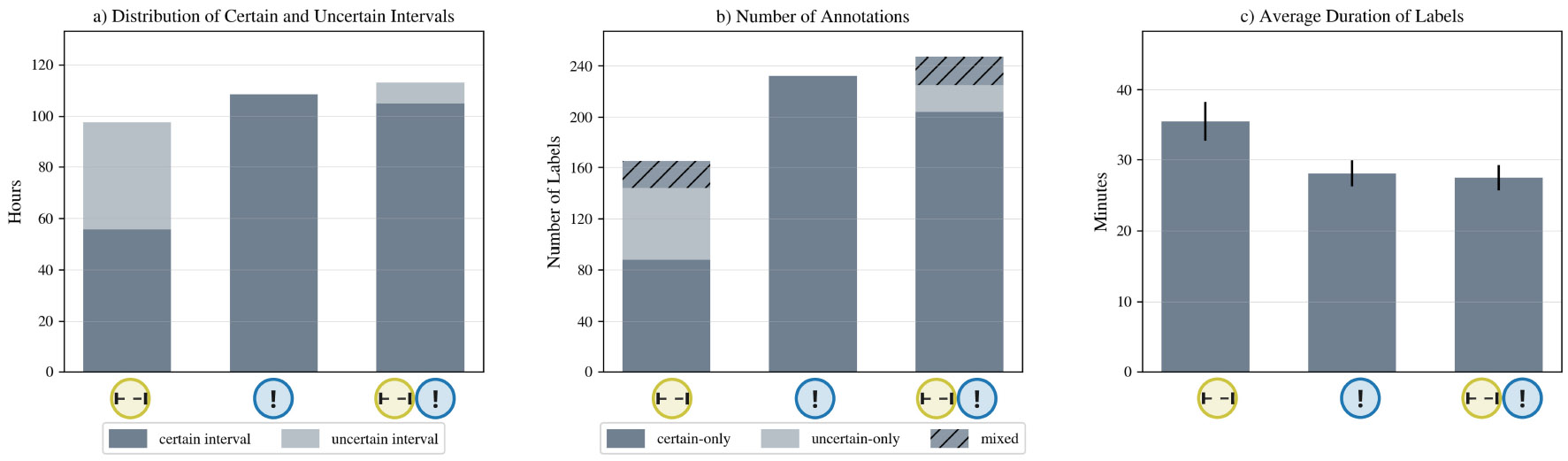
Across the three conditions, the distribution of certain vs. uncertain labels (in hours), the number of annotations, and the mean length of each annotation.

**Fig. 6.**
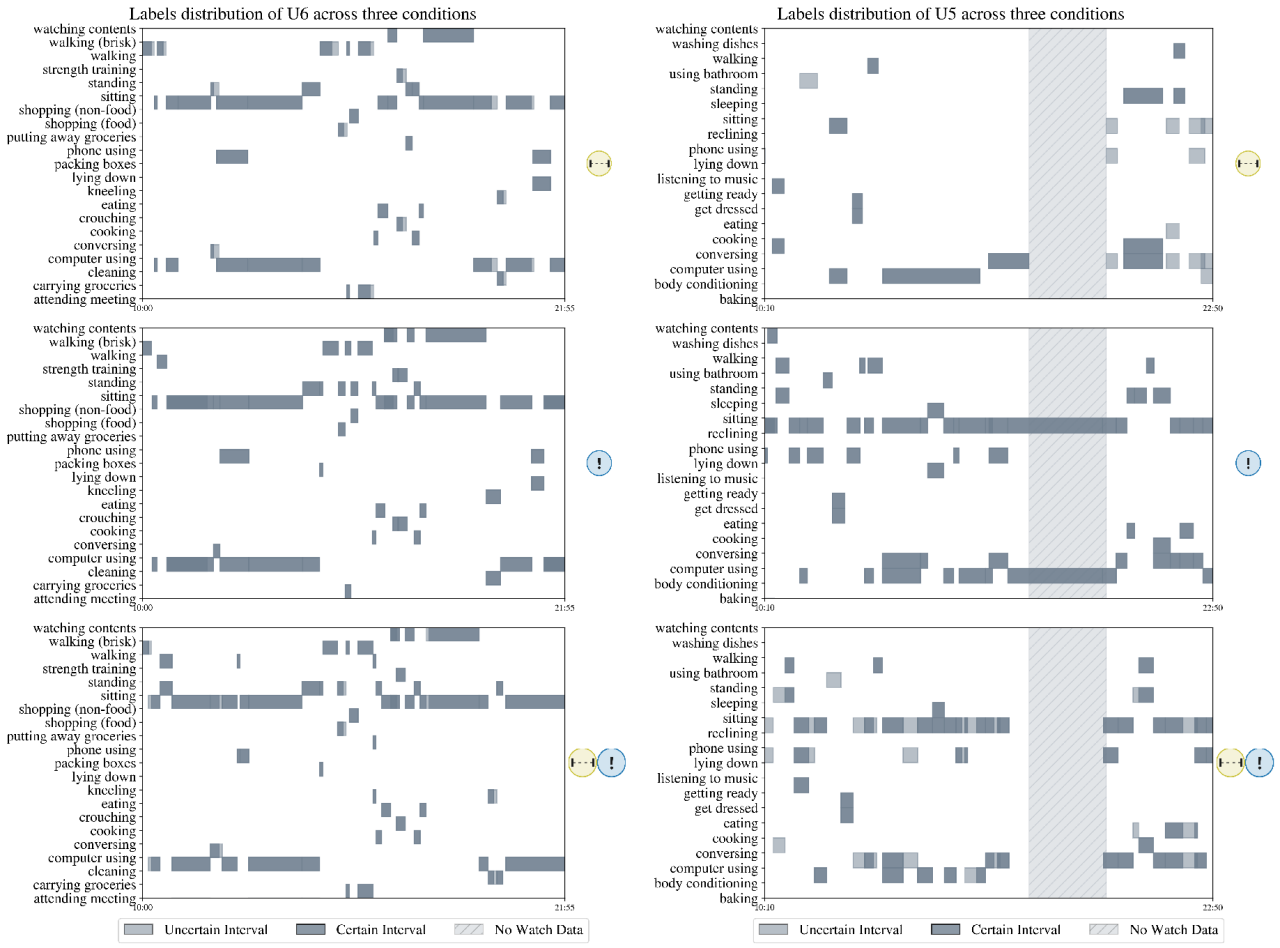
Label reporting across versions. Each subplot shows the temporal distribution of labels for an individual (U6 and U5). Participants annotated the same day using three different versions of ACAI. U6 labeled with relatively consistent content, density and duration across and conditions; U5, however, had noticeably sparser, longer labels for version 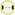 and more detailed, denser labels for version 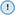 and 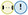.

Participants also commented that the label suggestions reduced their recall burden and helped them better triangulate information on the interface: *“Pre-filled activities give you an idea as to what you were doing and you could recall according to the step count as well the heart rate. The step count is high and I can see [the system suggested] standing or walking, so maybe I was traveling somewhere” [P11]*. Participants also noted that label suggestions made them more aware of brief actions occurring within a longer activity, helping them identify when exactly these moments took place: *“I think that where I see the positive in it is that it makes you think that by this time [point to the suggestion] I finished doing something. So it kind of brings up the the possibility that I did move, that I did get out of the bed […]. Instead of being too broad [with my annotation]” [U7]*. The mean duration of the annotations in version 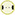 is longer than in both versions 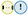 (Figures 5b and c), despite version 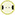 having fewer annotations overall. We show an example of this behavior in Figure 6.

The label suggestions from the system also influenced participant annotation, especially with regard to their annotation of uncertain intervals: *“I feel that they (the suggestions) are useful to recall as well, and I was more confident about what actually happened [sic.]” [U1]*. This bias explains why the distribution of “certain” vs. “uncertain” differed significantly between the two versions 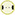 and versions 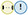 (*χ*^2^(1) = 33.61, *p* < .001). Although 44.1% of the data were annotated as uncertain in version 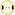, this percentage dropped to 7.6% in version 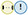 (see Figure 5a). Because version 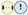 was highly rated and appreciated by our participants in Study 1, we decided to use it for our longer, in-the-wild deployment of ACAI.

### 5.2 Measuring the criterion validity of the collected labels (RQ2)

We measured the criterion validity of the collected labels across three conditions–each a different annotation mechanism (24PAR, ACT24, and ACAI)–with two different approaches using the objective accelerometer data from the thigh sensors.

#### 5.2.1 Activity intensity level.

We used the accelerometer data from the GT9X Link thigh sensor to compute Monitor-Independent Movement Summary (MIMS) units^2^ with one-minute epochs [44]. MIMS is a unit used to quantify physical activity from raw accelerometer data, designed to be comparable across different devices and studies. We hypothesized that higher intensity of self-reported activity correlate with higher MIMS unit [82]. We categorized the labels collected from the 24PAR interview, the ACT24 (the digital version of the 24PAR), and our annotation interface (ACAI) into four different activity intensity levels (*Sedentary, Light, Moderate* and *Vigorous*) following the guidelines from the Compendium of Physical Activity (CPA) [36]. We applied a linear mixed effects model with random intercept and interaction:

MIMS∼IntensityLevel∗condition+(1∣condition)+(1∣subject)

Here, *MIMS* is the MIMS unit calculated from one minute of accelerometry data from the thigh. *IntensityLevel* is one of the categories obtained from participant annotations using the CPA. We coded *IntensityLevel* into ordinal values. The variable *condition* is ACT24, 24PAR or ACAI, and *subject* is the participant ID.

**Fig. 7.**
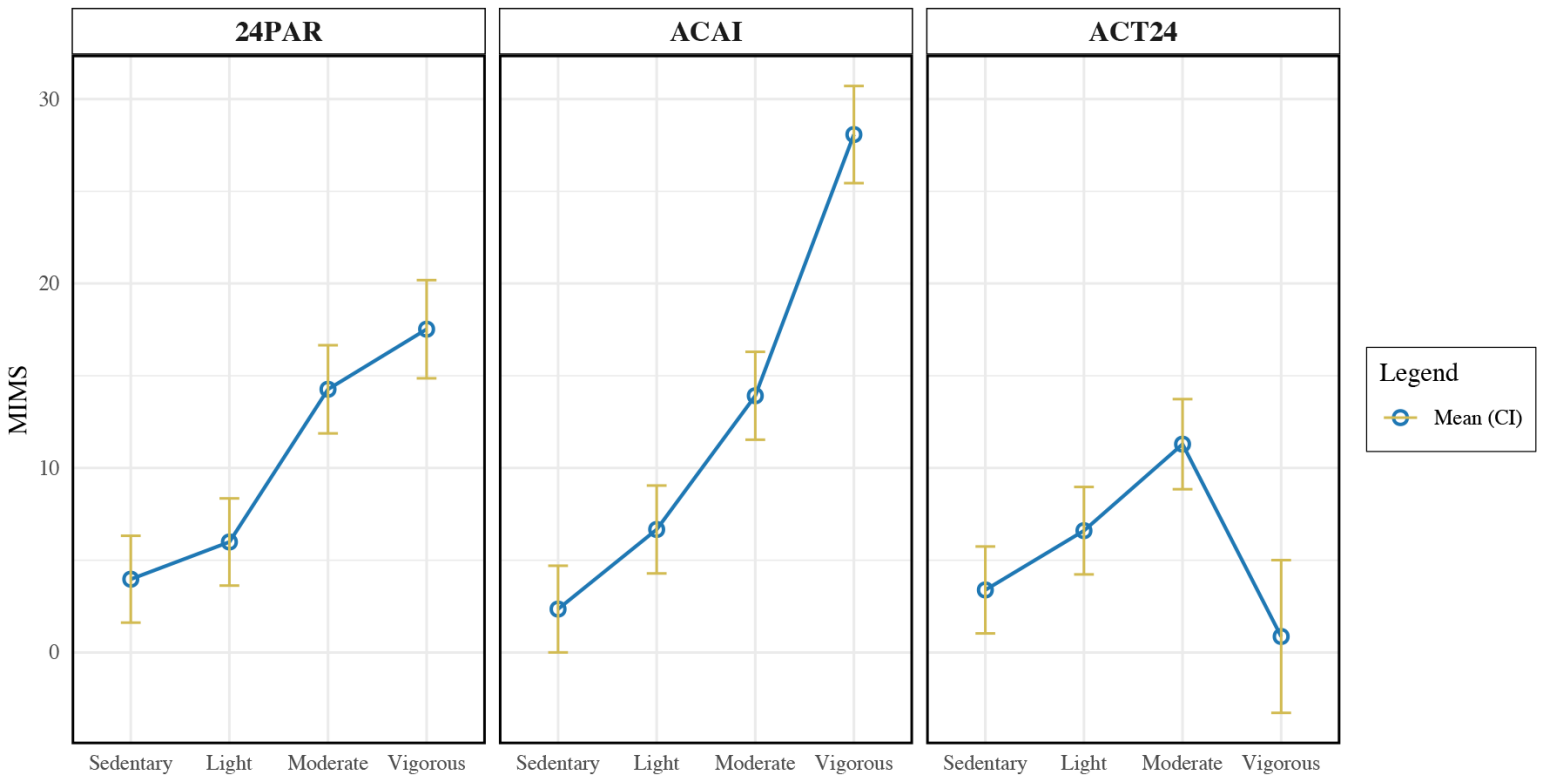
Annotated activity intensity level in three conditions (ACAI, 24PAR and ACT24) and the intensity level measured by the thigh sensor. The ACAI condition shows clear distinctions in MIMS unit between Sedentary, Light, Moderate, and Vigorous activity levels. The 24PAR condition shows statistically significant differences between Sedentary/Light vs. Moderate/Vigorous activities. The ACT24 condition, however, shows less plausible and weaker correlations – such as vigorous activities being associated with lower MIMS values than sedentary, light, or moderate activities – indicating that this annotation method likely introduced substantial errors.

The intensity level measured using the thigh sensor (MIMS unit) showed a significant positive trend as the annotated intensity level increased (*β* = 4.16, *p* < .001). In the ACAI condition, the relationship between intensity level and MIMS unit was significantly stronger, with a positive interaction coefficient of *β* = 1.95 (*p* < .001). This suggests a greater increase in MIMS unit as intensity increases in the ACAI condition compared to the reference condition (24PAR). In the ACT24 condition, the relationship between intensity level and MIMS unit is weaker, with a negative interaction coefficient of *β* = −0.77 (*p* < .001). As the annotated intensity increases in the ACT24 condition, the MIMS unit increases less than in the 24PAR condition. Figure 7 shows the interaction between the annotated intensity level and thigh sensor MIMS units across participants. This finding suggests that the annotations generated using ACAI yield the most distinct and plausible MIMS patterns across the four intensity levels. Specifically, the MIMS values increased in a consistent and expected manner (sedentary < light < moderate < vigorous), indicating a strong alignment between ACAI labels and sensor-derived activity intensity. In contrast, while 24PAR showed some separation between low and high intensity levels, ACT24 displayed weak or highly implausible trends—for example, vigorous activity sometimes corresponded to lower MIMS values than sedentary. This may be due to misaligned timestamps in the annotations generated by ACT24.

#### 5.2.2 Detected locomotion.

To further validate the self-report recalls, we compared the self-reports to the output of a classification system using the raw thigh sensor accelerometer data. Prior works have shown random forest (RF) classifiers work well for the task of human activity recognition [25, 40, 77]. We trained an RF model and evaluated on a subset of thigh accelerometer data from the PAAWS dataset simulated free-living and laboratory (SimFL+Lab) data [84]. We trained the model to predict five activity categories: *Running, Walking, Standing, Sitting*, and *Lying Down*. On the PAAWS dataset the RF achieved a weighted F1 score of 0.87 ± 0.03 on average across both thighs using a leave-one-participant-out cross validation. We included details about the implementation as well as the class-wise evaluation of the model in Appendix C.

We combined the “Sitting” and “Lying down” labels into “Sitting/Lying down” and “Walking” and “Running” labels into “Walking/Running” to be consistent with the postures recorded in ACT24 and 24PAR protocols. We list the F1 score between the HAR model predictions and the annotations across three conditions in Table 4. For each category (“Sitting/Lying down,” “Standing,” “Walking/Running”) we filtered out the segments where participants annotated the category, and the segments where the RF model predicted the category to calculate the F1 score. For posture A, we define TP (true positive) as the number of minutes when both the participants and the RF model annotate posture A, FP as the number of minutes when participants annotate posture A but the RF model predicts a different posture, and FN as the number of minutes when the RF model predicts posture A but the participant annotates a different posture. The F1 score is calculated as follows:

$$Precision=\frac{TP}{TP+FP};Recall=\frac{TP}{TP+FN};F1=2\times \frac{Precision\times Recall}{Precision+Recall}$$

Overall, the annotations collected via ACAI demonstrated higher agreement with HAR model predictions across all activity categories than both the 24PAR and ACT24 annotations. When analyzing only intervals labeled as certain by participants, the agreement rate for “Walking/Running” activities was slightly higher than when incorporating both certain and uncertain intervals in the analysis.

**Fig. 8.**
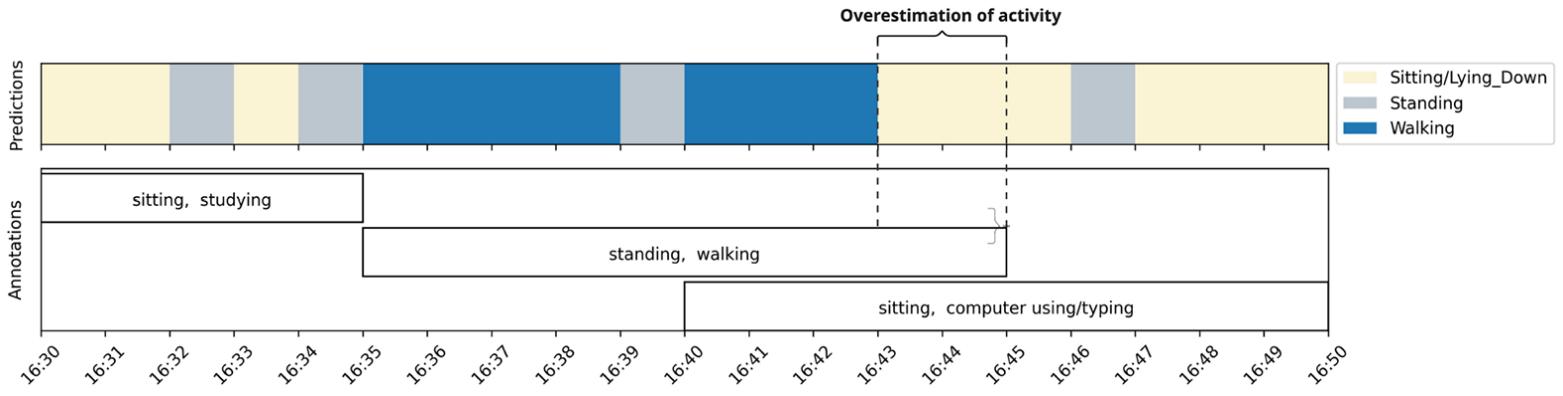
An example from a participant in our study demonstrating how an ACAI design limitation can lead to overestimation of activity duration. Because we only allow participants to annotate in five-minute blocks with no option for categorical uncertainty, the participant created overlapping labels, overestimating the duration of both the “walking” label (by 2 minutes) and the “sitting” label (by 3 minutes).

**Fig. 9.**
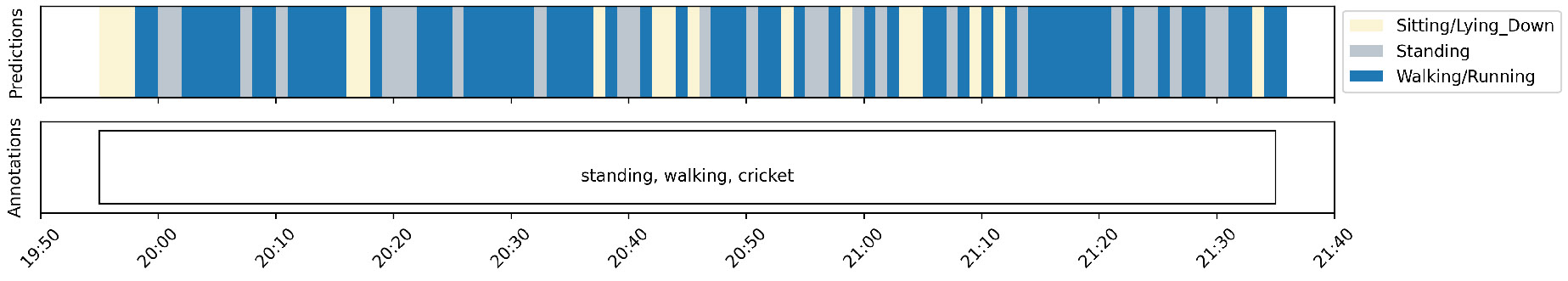
Another example from a participant in our study demonstrating how an ACAI design limitation can lead to overestimation of duration of some activities. In this case, participants engaged in a complex activity (playing cricket), which included fast-changing postures. Instead of creating different labels for “walking, playing cricket” and “standing, playing cricket,” they merged the entire period as “standing, walking, playing cricket.”

Despite annotations from ACAI showing higher agreement rate with the HAR model compared to ACT24 and 24PAR, the overall agreement rate was lower than we expected, specifically for “Walking/Running.” There are two main reasons for this low agreement rate: first, we noticed that participants tend to overestimate the duration of their annotations. The HAR model made a prediction once every minute, but in 24PAR, ACT24 and in ACAI, participants annotated in five-minute blocks. Therefore, if a participant took a walk from 16:35-16:43, they would annotate the entire block from 16:35-16:45 as “walking” (see Figure 8). The second cause of low agreement resulted from allowing participants to express temporal uncertainty (*when* an activity occurred) but not categorical uncertainty (*what* specific activity occurred). This design differs from both the 24PAR and ACT24, which permit participants to annotate proportional activity distribution (e.g., 95% sitting and 5% walking) within a time segment. This design limitation in ACAI resulted in situations such as the one we demonstrate in Figure 9: A participant spent two hours playing cricket, which was a complex activity involving multiple fast-changing postures. Unable to recall the precise timing of these postures, the participant created one “standing, walking, playing cricket” label that lasted for the entire two-hour period rather than specifying discrete time intervals for each posture. This design limitation subsequently contributed to the systematic overestimation of some labels, resulting in lower-than-expected agreement rates when compared to the HAR model’s predictions.

### 5.3 Feasibility of annotating data in free-living settings (RQ3)

We present exploratory findings from the feasibility portion of the second study (Study 2.2), including the quantitative and qualitative results.

#### 5.3.1 Quantitative results.

**Fig. 10.**
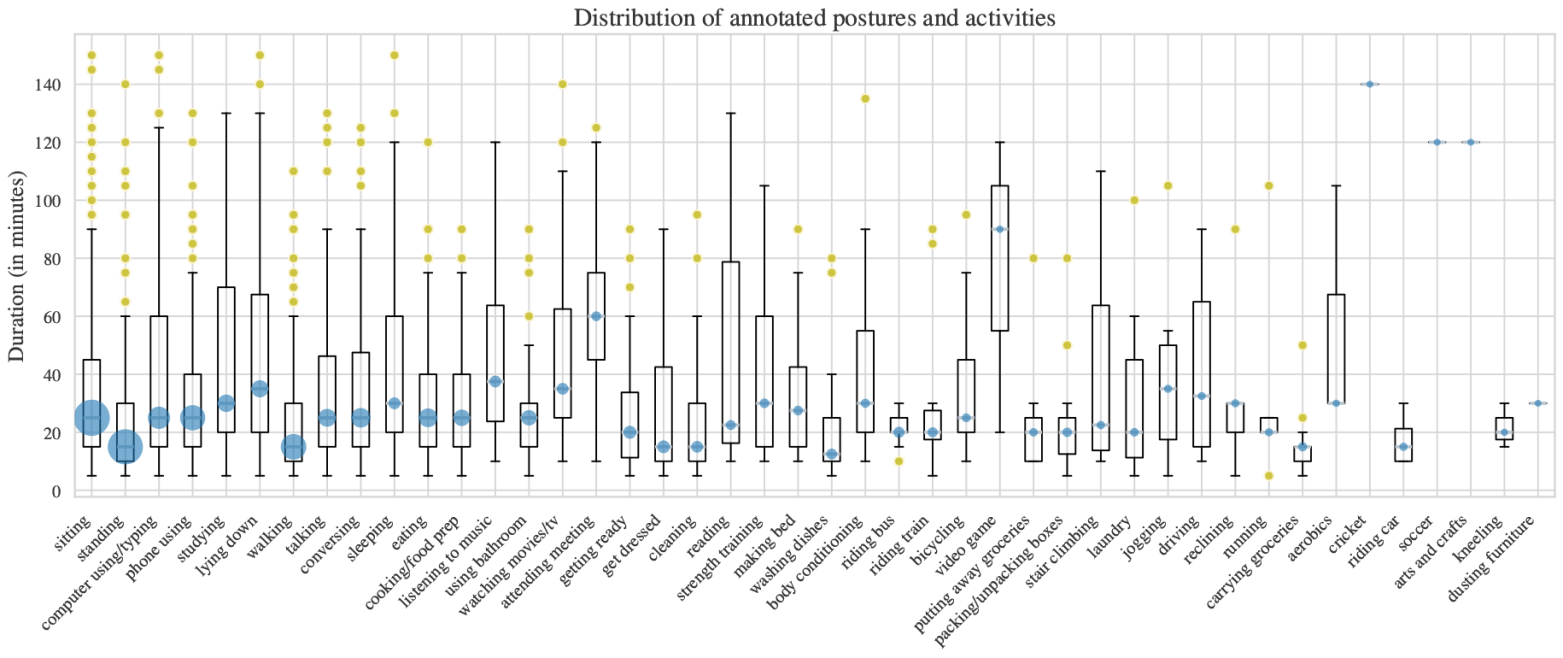
Distribution of annotated activities/postures in Study 2.2. The box plot shows the distribution of duration for each activity/posture. The blue circle denotes the median duration, and the size of the circle denotes the number of annotations for each activity/posture.

During Study 2.2, over the course of seven days, we collected 93,905 min of annotated data, with 92,620 min (98.5%) labeled as certain and 1,265 min (1.5%) labeled as uncertain. On average, we collected 14.7 hours of annotated data per day (excluding sleep). We collected 3,004 annotations, each with mean duration of 21.7 min (excluding sleep). Among all the annotations, 311 of them (10.4%) have only activity labels, none (0%) have only posture labels, and 2,693 (89.6%) contain both posture and activity labels. We show the distribution of annotated activities in Figure 10. Overall, we collected 45 unique activity and posture labels; including longer bouts of activities like “attending meeting” (*M* = 62.6 min, *SD* = 32.3) or “video game” (*M* = 76.7 min, *SD* = 41.1) and shorter bouts of activities like “carrying groceries” (*M* = 15.8 min, *SD* = 11.4), or “washing dishes” (*M* = 19.2 min, *SD* = 9.3). The median daily annotation time was 10.8 min (SD = 9.1). In comparison, the median duration of a 24PAR interview in Study 2.1 was 23.5 min (*SD* = 10.3)^3^. Participants used the application once daily on 55.8% of days and multiple times daily on 44.2% of days throughout the seven-day study period. The mean response rate for *μ*EMA was 68.4% (*SD* = 14.7).

We used a repeated-measures ANOVA to test whether there were changes in participants’ reporting patterns over time. We found no changes in time taken to annotate (*F*[7,89] = 0.62, *p* = 0.8), the number of annotations (*F*[7,89] = 0.97, *p* = 0.5), and the duration of individual annotations (*F*[7,89] = 0.77, *p* = 0.6) over time.

#### 5.3.2 Qualitative results.

To assess qualitative reactions to ACAI in Study 1 and Study 2, two authors carefully read each transcript from our field studies and independently performed open-ended inductive coding. The codes were generated and improved iteratively. We merged similar codes/themes and removed codes outside the scope of our research. The inter-rater agreement (Cohen’s kappa) after cleaning up the codes was *κ* = 0.87.

##### Perceived effort.

Participants identified perceived effort as the main factor affecting the usability of recall systems, including both interface interaction demands and scheduling flexibility. Ten out of 14 participants in Study 2 preferred digital recall interfaces over the 24PAR interview method, primarily due to scheduling flexibility (e.g., *“The interview is just you have to coordinate with somebody else and then you might be late and you might need to reschedule”* [P9]). The majority of participants in Study 2 (11 our of 14) preferred to annotate their activities at the end of the day. Four participants preferred the 24PAR interview approach, appreciating that it eliminated manual annotation tasks (e.g., *“Obviously my favorite will be annotating it on the Zoom [call] because I don’t have to put any efforts to it”* [P8]). Among the digital options without human supervision, eight participants favored ACAI over ACT24, citing the ability to revise annotations as a key advantage (e.g., *“I think I could also edit it [the annotation] if I make a mistake. You know, I think sometimes you put the pressure on yourself. You’re like, I gotta remember every single part of my day [at once] where in this case, you’re doing it yourself so you can take time”* [P5]).

##### Perceived label accuracy.

Ten out of 14 participants perceived that the annotations using ACAI were more accurate compared to annotations in the 24PAR interviews and ACT24, mainly due to the contextual cues presented in ACAI interface (e.g., *“I think the one on my phone would be better because it is also showing you where you are or terms of location and it’s also showing your footsteps and your heart rate”* [P9]). Three participants did not perceive any differences in reliability across three conditions, and one participant believed the 24PAR facilitated the most reliable annotation because of the interviewers’ probing (e.g., *“When you are asked the questions, you tend to figure out the answer, like when [the interviewer] asked me, OK, what else you were doing for this time. So I got back into the memory and realized, oh, I was doing this”* [P1]).

During both Study 1 and 2, of the seven types of contextual cues presented on the interface, *μ*EMA responses and step count were often flagged as helpful for recalling activity: *“So my the data which I relied on most was my step counts because I know that if I’m walking, my step count is high”* [P7]; *“A voice note (μEMA) that is written [on the interface], like if I’m talking about some movie, then the conversation is actually noted there. So I can I know that I was conversing, I was walking and I was talking on the phone or something”* [P11]. For the other contextual cues, the preference varied among participants, based on their lifestyle and habits (e.g., mobility patterns or calendar and phone usage). For example, P4 mentioned the use of location during the recall process, saying *“I actually really like the location part because if I can’t remember what I’m doing, I’m like, oh, I’m at this location and I roughly know that this is the classroom, so I was probably studying while I was in this classroom.”* U7 said *“I would like phone usage. That would tell me when I was on my break. That’s how I see it [being used since] I used to be a server”*. Three participants noted that they did not use an online calendar so the calendar section did not provide useful reminders, with one saying *“I don’t usually use that [calendar] on my phone”* [U1].

##### Using uncertainty annotation.

Only five of 14 participants mentioned using the uncertainty annotation frequently. Four said that the uncertainty annotation was useful for annotating the transition periods between activities (e.g., *“It’s pretty useful. I have used it because there are it’s, you know, our days are not exactly discrete. There is a transition period where you know from getting ready to walking out the door and riding in the bus and even from getting up from the bed and going to the bathroom, there’s a transition period”* [P9]). Three participants mentioned that the uncertain annotation was useful at the beginning of the study, but became less useful as they were more conscious of their activities later in the study. For example, one said *“In the beginning, I use it because I wasn’t sure about my activities, but after the first day I was really sure and particular about the activity which I have been doing”* [P1].

## 6 DISCUSSION AND IMPLICATIONS FOR FUTURE WORK

The findings of our studies (related to the research questions) contribute to the existing literature and have implications for future work on context-assisted activity recall and highlight the need to design future human-in-the-loop activity recognition systems that require some end-user labeling of activity.

### 6.1 Design implications for future semi-automated activity recall systems (RQ1)

Study participants have difficulty accurately remembering the precise start and end times of their activities, which presents a significant challenge for systems that require end-user-labeled activity to support human activity recognition tasks [57]. Our ACAI system enables participants to incrementally label their own activities, aided by contextual cues and auto-generated suggestions, and where they are able to express uncertainty about activity transitions. Our usability studies revealed that incorporating uncertainty annotation did not increase perceived workload or annotation time, nor did it decrease system usability. Participants demonstrated a clear preference for modifying system-generated suggestions over creating annotations manually, with the semi-automated approach reducing annotation time and enhancing system usability. Interestingly, our findings indicated that combining these two design elements, uncertainty annotation and semi-automation, can introduce unexpected participant bias, where users report higher confidence in their annotations and consequently underutilize the uncertainty feature. These findings align with recent work suggesting that AI can affect users’ recall and perceived confidence [66, 114].

A significant concern reported by participants in both studies was the cognitive and physical burden associated with activity recall. While the uncertainty annotation feature helped reduce some of the cognitive load—by allowing participants to express ambiguity in their responses—it also introduced increased annotation time and interaction effort. In Study 1, results showed that effective interaction design could help reduce this physical burden and shorten annotation time.

Several participants in Study 2, expressed a preference for conversation-based recall over screen interactions. Recent research supports this direction. Voice-based interfaces have been explored to elicit activity labels more naturally from participants [16, 54, 94]. With the rise of large language models (LLMs) and growing interest in LLM-powered self-report systems [20, 65, 89, 108], there are promising opportunities to integrate conversational agents into ACAI to make activity recall easier [6, 75].

Moreover, part of the cognitive burden may result from ACAI not supporting the expression of categorical uncertainty. Although we initially considered supporting categorical uncertainty, internal testing revealed that it significantly increased annotation time and effort, so we did not include it in the final deployment. Potter et al. recently investigated how different visualizations of categorical and temporal uncertainty influence user perception and performance in activity timelines [83]. Their findings suggest that uncertainty encoding can affect how users engage with recall tasks. Future work should explore how to design such interactions in a way that supports multiple uncertainty expressions without increasing annotation effort.

### 6.2 Effects of retrospective recall on data validity (RQ2)

Researchers have increasingly adopted retrospective recall systems to collect ground truth labels from study participants as an alternative data collection approach. This methodology addresses limitations of traditional *in-situ* measurements such as EMA by reducing contextual biases [73, 81] and interruption burden [61]. Researchers have shown that context-assisted recall can increase accuracy when measuring moods and affects [86] as well as increase annotation consistency and reduce missing data for measuring activity [39, 103]. In this paper, we compare our proposed context-assisted activity annotation system, ACAI, with existing standards for activity recall. Our results show that ACAI significantly outperforms both the human-supervised recall method (24PAR) and the unsupervised digital recall method (ACT24), when evaluated against validated activity intensity units (MIMS) and a human activity recognition (HAR) model. Most participants also had higher perceived reliability and usability for ACAI as compared with 24PAR and ACT24.

We identified several design limitations that can affect annotation quality. Our decision to allow participants to annotate activities in five-minute intervals rather than minute-by-minute segments (with an uncertainty option) was based on previous research findings [103] that showed that participants found minute-by-minute annotation excessively tedious. This granularity, however, introduced unexpected challenges and participant biases: we observed that participants frequently overestimated the duration of certain activities, particularly “walking,” instead of using the uncertainty annotation feature when appropriate. This issue highlights the need for two improvements: (1) design modifications to minimize such overestimation tendencies, and (2) development of models that can work with labels that are slightly temporally misaligned. Recent research on weakly-supervised learning suggests datasets with imprecise timestamps might be used to train activity recognition models [3, 90, 91]. From prior work and the results of our study, however, we believe that it is impractical to expect participants in a research study to recall the timestamp and duration of their activities to the seconds/minutes. Instead, we envision a human-AI collaborative system where an adaptive AI model can correct and flag irregularities of human annotations. Over time, the model can adapt and learn about the participants’ daily habits and behaviors to make more accurate activity predictions and suggestions.

Future research should investigate the internal reliability of ACAI-collected annotation, specifically examining the efficacy of machine learning models trained on these annotations.

### 6.3 Towards building human-in-the-loop activity recognition systems (RQ3)

Systems that track personal habits and lifestyle patterns over time are invaluable for health informatics applications and can be integrated into health intervention frameworks that promote longevity and well-being [22, 24, 67]. Individual habits and activities, however, naturally evolve, necessitating human-in-the-loop systems where participants can continuously monitor their behaviors and refine model predictions to accommodate their changing lifestyle patterns [1, 2, 42]. The ACAI semi-automated system enables participants to track and annotate their activities through both real-time (*in-the-moment*) and retrospective (*after-the-fact*) mechanisms. During the seven-day study, participants consistently reported diverse activities with minimal attrition or data gaps. Our current implementation relies on heuristic models for activity suggestions and is adequate for investigating the properties of the interface we describe in this paper, but future iterations should incorporate on-device, real-time machine learning models that continuously adapt based on participant annotations [6, 105, 110]. Additionally, our interface currently displays predictions without explanations, which can frustrate users when encountering inaccuracies or lead them to develop misconceptions about system functionality. Recent research trends in activity recognition interfaces point toward systems that provide transparent, human-understandable explanations [51, 111] and enable users to adjust and train models according to their unique lifestyle patterns and preferences [64]; end-user annotation systems like ACAI will be required to conduct studies on such systems.

## 7 LIMITATIONS

Although our study demonstrated ACAI to be a promising solution for end-user annotation, it has some limitations. The first limitation is our small sample size, which skewed towards young adults under 30 years old, 83% of whom self-reported to be familiar with self-tracking tools. The sample size in our second study also skewed towards non-Hispanic Asian students, which limits the generalizability of our findings. Future research should validate the design principles of ACAI in other populations (e.g., older adults). The second limitation of our work is the short study duration. Many data collection studies only run for one week; however, future research should explore the use of our system in longitudinal studies to assess long-term compliance and data quality. The third limitation of our work is the battery limitation of the Pixel Watch 2. Due to the intensive collection of passive sensing data and *μ*EMA, the watch only ran 12–13 hours on a charge. This limited our ability to gather a complete 24-hour dataset, which is why we did not collect sleep-related data or labels. Another design limitation of our study is that we used fixed thresholds in the ACAI visualization panel for heart rate and step count based on age range; these values may not generalize well across individuals. This simplified design helped standardize feedback during the study, but future deployments could adopt more personalized strategies. For instance, thresholds could be dynamically adjusted using recent activity data from platforms like Google Fit, or initialized using self-reported age and fitness level acquired during onboarding. These approaches would improve scalability while ensuring thresholds remain suitable for all participants. Due to the scope of our study, we only assessed the criterion validity of the annotations collected using ACAI. Future research should assess whether the annotations and data collected from our system can be used to train personalized HAR models that can adapt over time. Lastly, our system design and user study setup aims to explore the usability and validity of ACAI for research studies that must collect data and measure activities when participants receive little or no personal benefit from the measurement. In a free-living, non-study scenario where participants do not receive financial incentives, and where they may not establish rapport with and have frequent check-ins from a research team, labeling quantity and quality might be impacted. If our proposed annotation system were integrated into an intervention system that provides meaningful feedback to participants, it might increase participant motivation to provide high-quality annotations.

## 8 CONCLUSION

In this paper, we introduce ACAI, a novel context-assisted activity annotation interface that allows participants to provide detailed activity labels and start-end times using context-cued self report and auto-suggested labels that end users can“fix;” the system also allows participants to express uncertainty about temporal boundaries. We conducted two user studies with 24 participants to evaluate three different system designs and to measure the ecological validity of our system. We quantitatively and qualitatively assessed different factors that affect system usability and data quality, and as a result recommend guidelines for future end-user annotation systems. Our field deployment demonstrated the potential of using our system to collect rich, naturalistic activity labels, which can potentially support further research into adaptive human activity recognition systems.

## Supplementary Material

supplemental material

## Figures and Tables

**Table 1.** Summary of participant demographics for both studies

	Study 1 (n=11)	Study 2 (n=14)
Age (years)	*M* = 24.5, *SD* = 3.34, range = 20-31	*M* = 23, S*D* = 3.03, range = 18-28
Gender	8 Female, 3 Male	3 Female, 11 Male
Race	8 Asian, 2 Caucasian, 1 Black	13 Asian, 1 Black
Familiarity with tracking software/devices*	2 Very familiar, 8 Somewhat familiar, 1 Not familiar at all	4 Very familiar, 6 Somewhat familiar, 4 Not familiar at all
Physical activity level**	1 Active, 4 Moderate, 6 Sedentary	5 Moderate, 9 Sedentary

* Responses to the question *“How familiar are you with tracking applications on wearable devices (e.g., fitness trackers, smartwatches)?”*.

** Categorized based on the responses to the questions *“How much time on a typical day do you spend doing moderate/vigorous physical activity?”*

**Table 2.** Interaction counts for each condition, across all participants. (NA = not applicable)

Interaction Type	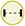	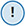	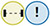
Add label	155	197	210
Change activity/posture of the suggestion	NA	214	172
Delete label	6	7	13
Edit label	45	10	16
**Scroll backward (manual)**	**268**	**122**	**43**
**Scroll forward (manual)**	**426**	**181**	**67**
Zoom in	6	1	3
Zoom out	13	12	13

**Table 3.** Linear mixed-effects regression model comparing intensity level of the anotated activity and the measurements from the thigh sensor across three conditions. 24PAR is the reference condition.

Predictor	Est.	p-value
IntensityLevel	4.16	<0.001 ***
condition (ACAI)	−3.38	0.07
condition (ACT24)	0.47	0.81
IntensityLevel × condition (ACAI)	1.95	<0.001 ***
IntensityLevel × condition (ACT24)	−0.77	<0.001 ***

**Table 4.** F1 score between the HAR model predictions and participants annotations in the three study conditions. ACAI (all) denotes all the annotations from the interface (both certain and uncertain labels). ACAI-Certain denotes only the certain annotations.

	24PAR	ACT24	ACAI (all)	ACAI-Certain
Sitting/Lying down	0.77	**0.84**	**0.87**	**0.87**
Standing	0.60	0.50	**0.68**	0.66
Walking/Running	0.30	0.22	0.45	**0.48**
Overall	0.68	0.78	**0.84**	**0.82**

**Fig. 11.**
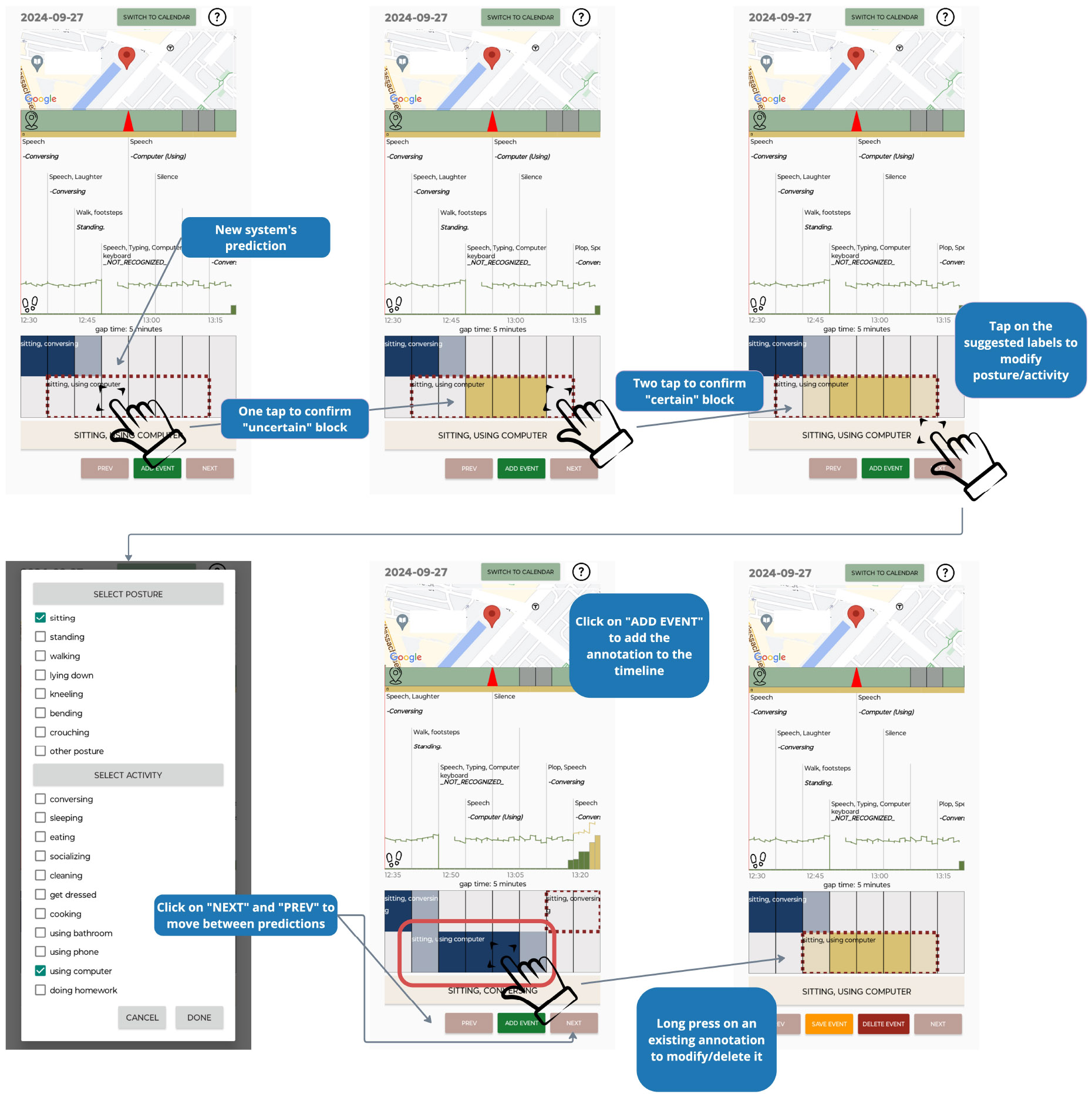
Interface interactions when using *ACAI*.

## Box

CCS Concepts: • **Human-centered computing → Empirical studies in ubiquitous and mobile computing**.

## Footnotes

1 https://cloud.google.com/speech-to-text.

2
https://www.rdocumentation.org/packages/MIMSunit/versions/0.11.2

3 We could not retrieve recall duration information from ACT24.

## A APPENDIX A: INSTRUMENTS

**Table 5.** List of item in the final usability survey participants filled out after trying all three versions of the interface; and how they related to the hypotheses.

Questions		Responses
Rank each contextual information based on their usefulness in recalling your posture/activity from most useful to least useful	—	Calendar events, Location, Step count, Heart rate, Self-report from smart watch, Memory, Ambient noises
The ability to indicate uncertainty gives me the flexibility while annotating	***H1***	
The ability to indicate uncertainty helps me annotate faster	***H1***	Strongly agree, Agree, Neither agree nor disagree, Disagree, Strongly disagree
The ability to indicate uncertainty makes annotating easier	***H1***	
The system suggestions on the timeline helps me annotate faster	***H2***	
The system suggestions on the timeline makes annotating easier	***H2***	

**Table 6.** List of invalidated instruments administered throughout the two studies. The daily burden survey is prompted through a participant’s phone at the end of every day of data collection. Other surveys are administered at the final sessions.

Scale	Purpose	Study 1	Study 2
SUS [12]	Measure usability of the system	X	X
NASA-TXL [15]	Measure mental/physical workload and the speed of a task	X	
Perceived burden survey [112]	Measure users’ perceived burden with the annotation task		X
Daily burden survey [41, 60]	Measure daily burden level and comfort with the smartwatch		X
Semi-structured interview	Collect qualitative feedback about the study and the usability of the system	X	X

## C APPENDIX C: HUMAN ACTIVITY RECOGNITION MODEL

The PAAWS dataset was collected from 252 participants engaged in 33 laboratory activities around a university campus. We used data from 38 participants^4^ to train the model. Since participants in our study could choose to wear the sensor on either their left or right thigh, we used the tri-axial acceleration data sampled at 80 Hz from an Actigraph GT9X-Link (the same model as the sensor used in our user study with similar placement) placed on the participants’ left and right thighs in the PAAWS dataset. For training and evaluation, we segmented the raw acceleration data into 60 s (4, 800 samples), non-overlapping windows and extracted 11 features from the accelerometer signal (66 features used in the feature vector). We implemented our RF model using sklearn v1.0.2, 1, 000 trees, and otherwise default settings.

Table 7 shows the list of features we extract from each axis of the thigh accelerometer signal. Table 8 shows the results of validating the random forest model on the PAAWS dataset.

**Table 7.** The eleven features we extracted from the thigh acceleration signal. Features were extracted from the raw signal and the fast Fourier transform (FFT) of the raw signal.

Feature No.	Description
1	Mean value of each axis (x, y, and z) and signal magnitude
2	Standard deviation (std) value of each axis (x, y, and z)
3	Mean deviation from mean of each axis (x, y, and z)
4	Median value of each axis (x, y, and z)
5	Minimum value of each axis (x, y, and z)
6	Maximum value of each axis (x, y, and z)
7	Number of peaks of each axis (x, y, and z)
8	Kurtosis of each axis (x, y, and z)
9	Skewness of each axis (x, y, and z)
10	Inner quartile range (IQR) of each axis (x, y, and z)
11	Area of signal magnitude

**Table 8.** The results of leave-one-participant-out cross validation of the random forest (RF) model on the PAAWS dataset. The trained model was used to validate the self-report labels in Study 2. Results are reported by thigh side (Right and Left) and by average.

Side	Lying_Down	Running	Sitting	Standing	Walking	Overall
**Right**	0.86 ± 0.05	0.56 ± 0.31	0.91 ± 0.03	0.79 ± 0.06	0.91 ± 0.02	0.87 ± 0.02
**Left**	0.86 ± 0.04	0.54 ± 0.32	0.91 ± 0.03	0.79 ± 0.06	0.91 ± 0.02	0.87 ± 0.03
**Avg**	0.86 ± 0.04	0.56 ± 0.32	0.91 ± 0.03	0.79 ± 0.06	0.91 ± 0.02	0.87 ± 0.03

## D APPENDIX D: PROMPTS FOR GENERATING LABEL SUGGESTIONS

We used the same prompt format developed by Le et al. [61], adapting the list of activities based on the Compemdium of Physical Activity 2024. The authors of the paper have ran the experiment with llama-3 8b and validated on two different dataset annotation schemes, CAPTURE-24 [17] and Pirsiavash et al. [79], and found that the model was able to categorize open-ended *μ*EMA responses to structured annotation schemes with low error rate (9-11%).

This is what the person reported doing: [participants’ self-report].

Map their self-report to one or more of the following labels:
    sleep,
    sitting,
    standing,
    lying,
    kneeling,
    bend over,
    ...
    [redacted the list of activities from the CPA]
If the self-report fits multiple labels, separate them with a backslash
(such as sitting/use computer, standing/cooking, ...).
If the self-report doesn't fit any of the labels, return 'others'.
Give a single answer (no explanation, limited prose).
Do not invent new labels.

## E APPENDIX E: DETAILS ABOUT ACTIVITY SUGGESTIONS ALGORITHM

To generate system suggestions for posture and physical activity, we followed two steps. First, we divided the tri-axial accelerometer data into non-overlapping distinct segments using binary segmentation and then mapped *μ*EMA to these periods to produce the final annotations. For the segmentation, the smartwatch estimated physical activity intensity using a real-time algorithm that measured the overall motion of the wrist based on accelerometer data. The smartwatch sampled raw tri-axial accelerometer data at 50 Hz and smooths the raw signal using a moving average filter with a window size of 0.5 s (filtered signal). For each axis, the app computes the area under the curve (AUC) $AUC_{t}=|raw_{t}-filtered_{t}|$ to compensate for the effect of gravity (DC offset for the axis) and calculates a 10 s summary of AUC by summing AUC values from the three axes to derive a physical activity summary unit [59-61]. The system runs a binary segmentation on the AUC signal (model = “rbf”, minimum window size = 5 min, penalty = 5) to split the data into different time periods. We called this segmentation $AUC_{seg}$.

Next, the system runs collected *μ*EMA responses through a large language model, llama-3 8b, to map the *μ*EMA self-report to a category in the Compendium of Physical Activity (CPA) 2024 [36]. We show details about the prompt in Appendix D. We called the list of mapped *μ*EMA responses and their corresponding timestamps *μEMA_lst*.

Finally, we map the AUC segments with its respective *μ*EMA responses. Time periods without any corresponding *μ*EMA responses are marked as “unknown” (or “?” when displayed in the interface). Algorithm 1 details the process of matching AUC segmentations to *μ*EMA responses.

**Table:**

Algorithm 1 Activity suggestion algorithm (*AUC_seg_*, *μEMA_lst*)
$$\begin{array}{cc} \; & \;1:\text{Initialize}\;final_{seg}\\ \; & \;2:\mathrm{for}\;seg\;\text{in}\;AUC_{seg}\;\mathrm{do}\\ \; & \;3:\;\mathrm{if}\;\text{no}\;\mu \text{EMA within}\;seg\;\mathrm{then}\\ \; & \;4:\;final_{seg}[seg]\leftarrow ‘‘\text{unknown}’’\\ \; & \;5:\;\mathrm{else}\;\mathrm{if}\;\text{only one}\;\mu \text{EMA within}\;seg\;\mathrm{then}\\ \; & \;6:\;final_{seg}[seg]\leftarrow \mu EMA\\ \; & \;7:\;\mathrm{else}\;\mathrm{if}\;\text{muliple}\;\mu \text{EMAs within}\;seg\;\mathrm{then}\\ \; & \;8:\;\text{Split}\;seg\;\text{into multiple segments based on}\;\mu \text{EMA timestamps}\\ \; & \;9:\;\text{Assign each of the sub-segments to the corresponding}\;\mu \text{EMA}\\ \; & \;10:\;\mathrm{end}\;\mathrm{if}\\ \; & \;11:\mathrm{end}\;\mathrm{for}\\ \; & \;12:\mathrm{return}\;final_{seg} \end{array}$$
