# Supplementary material for: A Context-Assisted, Semi-Automated Activity Recall Interface Allowing Uncertainty
Source: Proc ACM Interact Mob Wearable Ubiquitous Technol. Author manuscript; Available in PMC 2026 Jan 3. (PMC12758905; doi:10.1145/3770710)
Supplement: supplemental material [file NIHMS2119389-supplement-supplemental_material.zip › ACAI supp/interview_24PAR.pdf]

24PAR Interview Protocol

- If the interview is in person, set up a good environment for the interview
- If the interview is via Zoom, send the Zoom invitation to the participants

Interview Protocol

- **Introduction to 24PAR**
    - *Thanks for participating in this interview. My goal is to estimate how much time you spent doing both active and sedentary behaviors, and sleeping, in the last 24 hours). I want to create a complete picture of your last 24 hours.*
- **Ask for consent**
    - *Before we start the interview, I would like to ask for your oral consent for me to record the audio of this session. If you don't feel comfortable doing that, I will only take notes. Do you give me permission to record the audio of this interview?*
- **Begin Interview:**
    - **[note down the timestamp when the interview starts]**
    - *We are going to do a 24-hour recall together. I will be asking how you spent your time yesterday. Let's start with some questions about your work or school activities* **[ask the following questions and record the answers]**.
        - First, was the last 24 hours for you relatively typical or atypical in terms of your schedule and activities for a [Day of the week yesterday].
        - Did you work yesterday?
        - Did you go to school yesterday?
        - If you left your home, where did you go?
        - Do you have any stairs at your home? Where are they?
        - **[if went other places, ask about stairs at those places too]**
    - *Now, let's talk about your sleeping yesterday. By sleeping, I mean the time you go to bed at night until the time you wake up in the morning. You may not have been sleeping the whole time you were in bed. You may have been reading or watching TV for some of this time.*
        - What time did you go to bed last night? **[enter time]**
        - About how long were you in bed before you fell asleep? **[enter time]**
        - What time did you wake up this morning? **[enter time]**
        - Did you wake up during the middle of the night? If yes, for about how long? **[enter answer/time]**
        - What time did you go to bed the night before? **[enter time]**
        - About how long were you in bed before you fell asleep that night? **[enter time]**
        - What time did you wake up yesterday morning? **[enter time]**
        - In the last 24 hours, did you take any naps? **[enter answer] [If yes]** approximately what time did you nap and for how long?
    - *As we go through the rest of the interview, remember that there are no right and wrong answers. And you can add new information as you remember it. I am*

going to ask you about how you spent your time yesterday, starting when you got out of bed in the morning until you got into bed last night.
    - *In doing this, I want you to recall what you actually did, not what you usually do.*
- **Morning Recall**
    - *What time did you have lunch yesterday?* **[enter time. If no lunch was eaten, enter 12-noon, or a time that is relevant for the respondent, to define the morning time period (adjust as necessary for shift-workers).]**
    - *So, you got out of bed at **[time]** and ate lunch at **[time]**. Think about where you were and what you did during this **[number of hours between wake time and lunch]** hour period?* **[Record this information on the interview worksheet- ask follow up questions]**
        - You finish activity **[A]** at **[time]**. What did you do after [**A**]? (1) **[note down the answer *(activity B)*]**
        - **[Ask about the estimated timestamp of activity B]** (2) **[note down the answer]**
        - **[Ask about an estimated duration of the activity B]** (3) **[note down the answer]**
        - **[Ask about posture during activity B]** (4) **[note down the answer]**
        - **[Ask about the intensity of the activity B (define intensity level for participants if necessary)]** (5) **[note down the answer]**
            - *Vigorous intensity refers to a level of physical activity that is intense, requiring a substantial amount of effort and causing a significant increase in heart rate and breathing. Examples of vigorous activities include running, swimming laps, cycling at high speed, playing intense sports like basketball or soccer, and vigorous aerobic exercises such as high-intensity interval training (HIIT).*
            - *Moderate intensity refers to a level of physical activity that is moderately challenging and elevates heart rate and breathing, but doesn't reach the same level of intensity as vigorous activities. Examples of moderate-intensity activities include brisk walking, cycling at a moderate pace, water aerobics, dancing, gardening, and recreational sports like tennis or volleyball.*
            - *Sedentary activities involve activities or behaviors that require minimal physical effort and involve sitting or reclining for extended periods. Sedentary activities typically involve little to no physical movement and low energy expenditure. Examples of sedentary activities include watching television, working at a desk, using a computer, reading, playing video games, and driving.*
        - **[Ask about the location where activity B takes place (outdoor, house, workplace, …)]** (6) **[note down the answer]**
        - **[If the participant was traveling from place to place, ask about the mode of transportation and whether they took a stair in between activity A and B]** (7) **[note down the answer]**
        - **[If the participant was traveling from place to place, ask about the estimated/average speed/distance]** (8) **[note down the answer]**

- **[Multitasking - if the participant reported doing multiple tasks at the same time (e.g. reading and eating or cooking and child care)]** (9) **[note down the answer]**
  - If the activities happen concurrently, classify them as primary activity and secondary activities (e.g. If someone was watching television while walking on their treadmill, walking would be the primary behavior, television secondary.)
  - If the primary task switches from one behavior to another in a short period of time (e.g., reading the paper, checking email, and watching TV), get a “best estimate” of the [amount of time | percentage amount of time] spent in each (primary) behavior in the multitask period.
- **[Ask probing questions to refine information if appropriate]** (10)
  - For each exercise or sport reported, specifically ask for the overall time spent doing the activity, and then refine the estimate by asking to exclude break periods. Lower intensity warm-up or cool-down periods may be noted down separately if possible.
- **[Repeat (1 - 10) until you reach lunch time]**

- **Afternoon Recall**
  - *What time did you have dinner?* **[enter time. If no meal was eaten, enter 6:00PM, or a time that is relevant for the respondent, to define the afternoon time period (adjust as necessary for shift-workers).]**
  - *Remember, you ate lunch at **[time]** and had dinner at **[time]**. Think about where you were and what you did during this **[number of hours between lunch and dinner]** hour period?***[Record this information on the interview worksheet- ask follow up questions]**
    - You finish activity **[A]** at **[time]**. What did you do after [**A**]? (1) **[note down the answer *(activity B)*]**
    - **[Ask about the estimated timestamp of activity B]** (2) **[note down the answer]**
    - **[Ask about an estimated duration of the activity B]** (3) **[note down the answer]**
    - **[Ask about posture during activity B]** (4) **[note down the answer]**
    - **[Ask about the intensity of the activity B (define intensity level for participants if necessary)]** (5) **[note down the answer]**
      - *Vigorous intensity refers to a level of physical activity that is intense, requiring a substantial amount of effort and causing a significant increase in heart rate and breathing. Examples of vigorous activities include running, swimming laps, cycling at high speed, playing intense sports like basketball or soccer, and vigorous aerobic exercises such as high-intensity interval training (HIIT).*
      - *Moderate intensity refers to a level of physical activity that is moderately challenging and elevates heart rate and breathing, but doesn't reach the same level of intensity as vigorous activities. Examples of moderate-intensity activities include brisk walking, cycling at a moderate*

pace, water aerobics, dancing, gardening, and recreational sports like tennis or volleyball.

        - *Sedentary activities involve activities or behaviors that require minimal physical effort and involve sitting or reclining for extended periods. Sedentary activities typically involve little to no physical movement and low energy expenditure. Examples of sedentary activities include watching television, working at a desk, using a computer, reading, playing video games, and driving.*
    - **[Ask about the location where activity B takes place (outdoor, house, workplace, …)]** (6) **[note down the answer]**
    - **[If the participant was traveling from place to place, ask about the mode of transportation and whether they took a stair in between activity A and B]** (7) **[note down the answer]**
    - **[If the participant was traveling from place to place, ask about the estimated/average speed/distance]** (8) **[note down the answer]**
    - **[Multitasking - if the participant reported doing multiple tasks at the same time (e.g. reading and eating or cooking and child care)]** (9) **[note down the answer]**
        - If the activities happen concurrently, classify them as primary activity and secondary activities (e.g. If someone was watching television while walking on their treadmill, walking would be the primary behavior, television secondary.)
        - If the primary task switches from one behavior to another in a short period of time (e.g., reading the paper, checking email, and watching TV), get a "best estimate" of the [amount of time | percentage amount of time] spent in each (primary) behavior in the multitask period.
    - **[Ask probing questions to refine information if appropriate]** (10)
        - For each exercise or sport reported, specifically ask for the overall time spent doing the activity, and then refine the estimate by asking to exclude break periods. Lower intensity warm-up or cool-down periods may be noted down separately if possible.
    - **[Repeat (1 - 10) until you reach dinner time]**

- **Dinner Recall**
    - *Earlier you ate dinner at* ***[time]*** *and went to bed at* ***[time]***. **[Do not record activities occurring after 12-midnight]**
    - *Think about where you were and what you were doing in this* ***[number of hours between dinner time and bedtime]*** *hour(s) period?*
        - You finish activity **[A]** at **[time]**. What did you do after [**A**]? (1) **[note down the answer *(activity B)*]**
        - **[Ask about the estimated timestamp of activity B]** (2) **[note down the answer]**
        - **[Ask about an estimated duration of the activity B]** (3) **[note down the answer]**
        - **[Ask about posture during activity B]** (4) **[note down the answer]**

- **[Ask about the intensity of the activity B (define intensity level for participants if necessary)]** (5) **[note down the answer]**
    - *Vigorous intensity refers to a level of physical activity that is intense, requiring a substantial amount of effort and causing a significant increase in heart rate and breathing. Examples of vigorous activities include running, swimming laps, cycling at high speed, playing intense sports like basketball or soccer, and vigorous aerobic exercises such as high-intensity interval training (HIIT).*
    - *Moderate intensity refers to a level of physical activity that is moderately challenging and elevates heart rate and breathing, but doesn't reach the same level of intensity as vigorous activities. Examples of moderate-intensity activities include brisk walking, cycling at a moderate pace, water aerobics, dancing, gardening, and recreational sports like tennis or volleyball.*
    - *Sedentary activities involve activities or behaviors that require minimal physical effort and involve sitting or reclining for extended periods. Sedentary activities typically involve little to no physical movement and low energy expenditure. Examples of sedentary activities include watching television, working at a desk, using a computer, reading, playing video games, and driving.*
- **[Ask about the location where activity B takes place (outdoor, house, workplace, …)]** (6) **[note down the answer]**
- **[If the participant was traveling from place to place, ask about the mode of transportation and whether they took a stair in between activity A and B]** (7) **[note down the answer]**
- **[If the participant was traveling from place to place, ask about the estimated/average speed/distance]** (8) **[note down the answer]**
- **[Multitasking - if the participant reported doing multiple tasks at the same time (e.g. reading and eating or cooking and child care)]** (9) **[note down the answer]**
    - If the activities happen concurrently, classify them as <u>primary activity</u> and <u>secondary activities</u> (e.g. If someone was watching television while walking on their treadmill, walking would be the primary behavior, television secondary.)
    - If the primary task switches from one behavior to another in a short period of time (e.g., reading the paper, checking email, and watching TV), get a "best estimate" of the [amount of time | percentage amount of time] spent in each (primary) behavior in the multitask period.
- **[Ask probing questions to refine information if appropriate]** (10)
    - For each exercise or sport reported, specifically ask for the overall time spent doing the activity, and then refine the estimate by asking to exclude break periods. Lower intensity warm-up or cool-down periods may be noted down separately if possible.
- **[Repeat (1 - 10) until you reach bedtime]**

- **Next Morning Recall**

- *What time did you get up from bed this morning?* **[enter time]**
- *So you got out of bed at [time] this morning, and joined the meeting/ arrived at NU campus at* ***[time].*** *Think about where you were and what you did during this* ***[#]*** *hour period?* **[Record this information on the interview worksheet- ask follow up questions]**
  - You finish activity **[A]** at **[time]**. What did you do after [**A**]? (1) **[note down the answer *(activity B)*]**
  - **[Ask about the estimated timestamp of activity B]** (2) **[note down the answer]**
  - **[Ask about an estimated duration of the activity B]** (3) **[note down the answer]**
  - **[Ask about posture during activity B]** (4) **[note down the answer]**
  - **[Ask about the intensity of the activity B (define intensity level for participants if necessary)]** (5) **[note down the answer]**
    - *Vigorous intensity refers to a level of physical activity that is intense, requiring a substantial amount of effort and causing a significant increase in heart rate and breathing. Examples of vigorous activities include running, swimming laps, cycling at high speed, playing intense sports like basketball or soccer, and vigorous aerobic exercises such as high-intensity interval training (HIIT).*
    - *Moderate intensity refers to a level of physical activity that is moderately challenging and elevates heart rate and breathing, but doesn't reach the same level of intensity as vigorous activities. Examples of moderate-intensity activities include brisk walking, cycling at a moderate pace, water aerobics, dancing, gardening, and recreational sports like tennis or volleyball.*
    - *Sedentary activities involve activities or behaviors that require minimal physical effort and involve sitting or reclining for extended periods. Sedentary activities typically involve little to no physical movement and low energy expenditure. Examples of sedentary activities include watching television, working at a desk, using a computer, reading, playing video games, and driving.*
  - **[Ask about the location where activity B takes place (outdoor, house, workplace, …)]** (6) **[note down the answer]**
  - **[If the participant was traveling from place to place, ask about the mode of transportation and whether they took a stair in between activity A and B]** (7) **[note down the answer]**
  - **[If the participant was traveling from place to place, ask about the estimated/average speed/distance]** (8) **[note down the answer]**
  - **[Multitasking - if the participant reported doing multiple tasks at the same time (e.g. reading and eating or cooking and child care)]** (9) **[note down the answer]**
    - If the activities happen concurrently, classify them as primary activity and secondary activities (e.g. If someone was watching television while walking on their treadmill, walking would be the primary behavior, television secondary.)

            - If the primary task switches from one behavior to another in a short period of time (e.g., reading the paper, checking email, and watching TV), get a “best estimate” of the [amount of time | percentage amount of time] spent in each (primary) behavior in the multitask period.
        - **[Ask probing questions to refine information if appropriate]** (10)
            - For each exercise or sport reported, specifically ask for the overall time spent doing the activity, and then refine the estimate by asking to exclude break periods. Lower intensity warm-up or cool-down periods may be noted down separately if possible.
        - **[Repeat (1 - 10) until you reach meeting time]**
- **Indicate the reliability of the interview before you end the call**
    - *Do you think this recall is reliable?* **[Read through the summary to the participants]** *Is there anything you would want to add to the summary?* **[if yes, record information on the worksheet, ask follow up questions if necessary, and repeat this step]**
    - *Can you rate your level of effort in doing this recall from very low, low, moderate, high to very high?*
    - *If you think the recall was unreliable, please comment why:* **[record the participant answers]**
- **End this part of the interview**
    - **[note down the timestamp when the interview ends]**
    - *Thank you so much for participating in the interview!*
